# An earth-abundant bimetallic catalyst coated metallic nanowire grown electrode with platinum-like pH-universal hydrogen evolution activity at high current density†

**DOI:** 10.1039/d0sc00754d

**Published:** 2020-03-24

**Authors:** Sahanaz Parvin, Ashwani Kumar, Anima Ghosh, Sayan Bhattacharyya

**Affiliations:** Department of Chemical Sciences, Centre for Advanced Functional Materials, Indian Institute of Science Education and Research (IISER) Kolkata Mohanpur-741246 India sayanb@iiserkol.ac.in

## Abstract

A self-supported and flexible current collector solely made of earth-abundant elements, NiCo layered double hydroxide (LDH) wrapped around Cu nanowires (Cu-Ws) grown on top of commercially available Cu mesh (Cu-m), outperforms the benchmark 40 wt% Pt/C in catalyzing the electrochemical hydrogen evolution reaction (HER). The Cu-m/Cu-W/NiCo-LDH cathode operates both in acidic and alkaline media exhibiting high turnover frequencies (TOF) at 30 mV (0.3 H_2_ s^−1^ in 1 M KOH and 0.32 H_2_ s^−1^ in 0.5 M H_2_SO_4_, respectively) and minimal overpotentials of 15 ± 6 mV in 1 M KOH and 27 ± 2 mV in 0.5 M H_2_SO_4_ at −10 mA cm^−2^. Cu-m/Cu-W/NiCo-LDH outperforms the activity of 40 wt% Pt/C that needs overpotentials of 22 and 18 mV in 1 M KOH and 0.5 M H_2_SO_4_, respectively. With a tremendous advantage over Pt/C in triggering proton reduction with fast kinetics, similar mass activity and pH-universality, the current collector demonstrates outstanding operational durability even at above −1 A cm^−2^. The high density of electronic states near the Fermi energy level of Cu-Ws is found to be a pivotal factor for efficient electron transfer to the NiCo-LDH catalyst. This class of self-supported electrodes is expected to trigger rapid progress in developing high performance energy conversion and storage devices.

## Abstract 2

A flexible self-supported electrode made of earth-abundant elements, NiCo layered double hydroxide wrapped around Cu nanowires grown on Cu mesh, outperforms the benchmark 40 wt% Pt/C in catalyzing electrochemical hydrogen evolution reaction.

## Graphical abstract

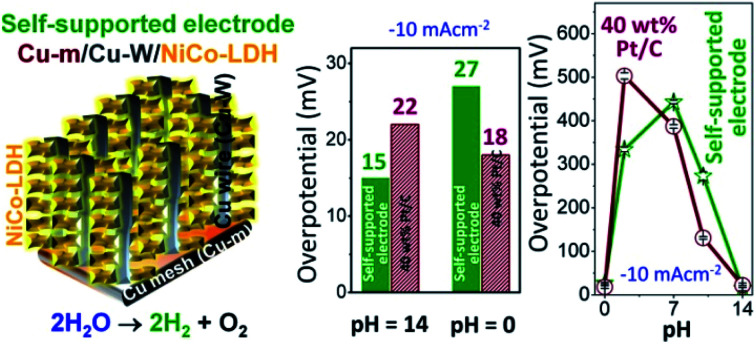

## Introduction

The potential of hydrogen as a sustainable energy carrier is indisputable in order to drive the future global demand for terawatt energy.^1^ One of the winsome avenues for accomplishing the technology of hydrogen conversion and storage is water electrolysis, especially that driven by solar- or wind-derived electricity.^2,3^ In electrochemical water splitting, the 2e^−^ hydrogen evolution reaction (HER) is a vital half-reaction besides the more sluggish 4e^−^ oxygen evolution reaction (OER).^4^ Real-life applications demand high-performance, durable and cheap catalysts to lower the steep activation energy barrier for driving the OER and HER. To date, Pt is the most well-known HER catalyst because of its undisputed predominance in catalyzing reactions with fast kinetics, minimal overpotential and high exchange current density.^5^ Nonetheless, the low elemental abundance of Pt leads to high costs in addition to its poor durability, which makes large-scale hydrogen production elusive. Various earth-abundant alternatives such as NiFe supported on mesoporous substrates,^6^ Ni–Mo alloys,^7^ MoS_2_,^8^ MoC–Mo_2_C nanowires,^9^ CoP on Ni_5_P_4_,^10^ or PANI,^11^ NiO/Ni/carbon nanotubes,^12^ and B/N co-doped graphene nanotubes,^13^ Cu_3_P nanoarrays,^14^ NiMoS_4_/Ti mesh,^15^ and Zn_0.08_Co_0.92_P/Ti mesh,^16^ have been identified as promising HER catalysts. In spite of an impeccable HER performance of many of these catalysts, a Pt-like activity has never been achieved. In very few instances when the catalysts have reached or bettered the Pt-limit, inclusion of other high cost and low abundant elements such as Ru and Ir becomes inevitable, for example, Ru/W_0.62_(N_0.62_O_0.38_)@C,^17^ RuP,^18^ Ru–Co,^19^ CoRu,^20^ RuM/CQDs,^21^ Ir@3D-organic networks,^22^ hollow carbon sphere-confined Ru nanoparticles (HCRNs),^23^ and Ru/PC^24^ in alkaline pH, RuPt,^25^ Pt-MXene,^26^ IrHNC^27^ in acidic pH and Ru@C_2_N with pH-universal activity.^5^

The need of the hour is to develop an earth-abundant pH-universal HER catalyst that can trigger proton reduction with Pt-like fast kinetics and the least possible overpotential. A rejuvenated self-supported electrode is even more attractive as it allows skipping polymeric binders that block catalytically active sites,^28^ and avoiding additional current collectors such as the glassy carbon electrode (GCE),^29^ carbon fiber paper (CFP),^30^ carbon cloth,^31^ and Ni or Cu foam.^32–34^ Rational design of self-supported electrodes is critically dependent on a fairly large exposed surface, enhanced mass diffusion and efficient electron transport with futuristic applications towards wearable and flexible energy devices.^35^ A few developments in this area are “pencil-on-paper” piezoresistive sensors,^36^ Ni-coated paper electrodes,^7^ hybrid electrocatalyst supported on Ni foam,^37^*etc.* Instead of using CFP or metal foams which are prone to air oxidation,^38^ pragmatic options such as electrically conducting and mechanically robust current collectors made of first row transition metals with high reduction potential can be considered. A suitable choice is earth-abundant copper whose native metallic form is commonly used for electrical wiring and does not react immediately with water but gradually gets oxidized by atmospheric oxygen to its weathered form Cu_2_CO_3_(OH)_2_, a greenish coating commonly observed over famous copper architectures namely the Statue of Liberty and Berliner Dom. Thus, being resistant to acidic and alkaline corrosion, copper substrates bring about the perfect solution under reductive bias for the electrochemical HER by concomitantly offering high electrical conductivity and mechanical robustness. Our self-supported electrode design starts from commercially available Cu mesh (Cu-m), onto which Cu nanowires (Cu-Ws) are grown by subsequent chemical oxidation and electrochemical reduction. When Cu-Ws are shelled with NiCo-LDH nanosheets, a flexible hybrid cathode Cu-m/Cu-W/NiCo-LDH is fabricated that outperforms the state-of-the-art 40 wt% Pt/C electrode across the entire pH range besides having exceptional durability at very high current densities, ideal for commercial applications. In fact a Cu nanowire core shelled with CoFe-LDH was previously explored by Yu & coworkers albeit the nanowires were supported on Cu foam, with moderate achievable success.^39,40^ The finesse of our approach first lies in using Cu-m as the substrate whose woven like porous structure helps in better release of gas bubbles and mass diffusion of the reactant and product. Secondly, our choice of metal combination in the LDH is rationalized by two facts: (i) minimal interaction of the 3d orbital of Ni and Co with the H 1s orbital assists the facile desorption of H_2_ from these two metal centers; and (ii) the first step of hydrogen evolution *i.e.* water dissociation followed by OH^−^ desorption becomes facile at the Ni and Co active sites due to the absence of any overlap between the Ni/Co 3d orbital and O 2p orbital.^41,42^ Other bimetallic combinations have subservient synergism, validated by several control experiments. Our ingeniously designed self-supported electrode helps to achieve the industrial demand of −1 A cm^−2^ at low overpotential with high operational durability, thus promoting the chances for commercialization.

## Results and discussion

**Fig. 1:**
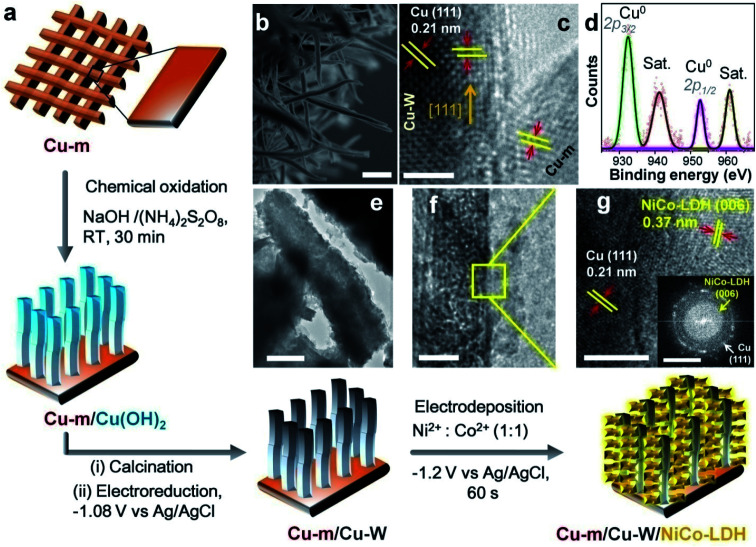
Fabrication and characterization of Cu-m/Cu-W/NiCo-LDH. (a) Schematic illustration of the fabrication of the Cu-m/Cu-W/NiCo-LDH electrode. (b) Cross-sectional FESEM image of the Cu-m/Cu-W substrate (scale bar 1 μm). (c) TEM image showing the interface between Cu-m and Cu-Ws (scale bar 2 nm). (d) Fitted XPS spectrum of the Cu 2p level from Cu-m/Cu-Ws. The open circles represent the experimental data and the best fit is indicated by the solid line. Sat. denotes the satellite peaks. (e, f) Low (scale bar 200 nm and 20 nm) and (g) high resolution (scale bar 5 nm) TEM images of Cu-m/Cu-W/NiCo-LDH. The inset shows the corresponding SAED pattern of the electrode (scale bar 5 nm^−1^).

As schematically illustrated in Fig. 1a, chemical oxidation of Cu-m was performed in an alkaline solution containing (NH_4_)_2_S_2_O_8_ where S_2_O_8_^2−^ ions are reduced to SO_4_^2−^ at the cost of Cu^0^ getting oxidized to Cu^2+^. The as-prepared Cu(OH)_2_ nanowires were converted to CuO nanowires grown on Cu-m by calcination at 180 °C, which were further reduced to face-centered cubic Cu-Ws by electrochemical reduction at −1.08 V in 1 M KHCO_3_; the phase transitions were monitored by powder X-ray diffraction (PXRD, Fig. S1†). The 4–5 μm long Cu-Ws have a diameter of 140 ± 10 nm, determined by cross-sectional field emission scanning electron microscopy (FESEM, Fig. 1b). When NiCo-LDH is electrochemically deposited on the Cu-m/Cu-W electrode by chronoamperometry at −1.2 V, the nanowire diameter increases to 163 ± 10 nm (Fig. S1d†), suggesting an average LDH thickness of ∼10 nm wrapped around Cu-Ws. High-resolution transmission electron microscopy (HRTEM) shows the (111) exposed facets of Cu-m and Cu-Ws, and the latter's growth direction is along [111] (Fig. 1c). X-ray photoelectron spectroscopy (XPS) analysis of the Cu 2p level provides evidence of the metallic state of Cu-Ws at binding energies of 932.4 eV (2p_3/2_) and 952.7 eV (2p_1/2_), whereas the shake-up satellite peaks at 941.2 and 961.1 eV show the presence of a surface thin layer of CuO, which however could not be envisaged from the core level positions (Fig. 1d).

High-angle annular dark-field scanning TEM (HAADF-STEM) mapping and elemental line scan provide evidence of the uniform wrapping of LDH nanosheets over the vertical Cu-Ws (Fig. S2†), also corroborated by energy dispersive X-ray spectral (EDS) mapping coupled with FESEM (Fig. S3†). The interconnected LDH nanosheets are vertically grown on Cu-Ws (Fig. 1e and f), ensuring maximum available sites of the NiCo-LDH catalyst. The lattice spacings of 0.21 and 0.37 nm correspond to the (111) reflection of metallic Cu and the (006) plane of the hexagonal unit cell of NiCo-LDH (JCPDS 40-216), respectively (Fig. 1g), corroborated by the selected area electron diffraction (SAED) pattern (Fig. 1g inset). The LDH structure consists of Ni^2+^, Co^2+^ and Co^3+^,^43^ discerned from the binding energies of 854.1, 781.0 and 779.4 eV in the XPS 2p_3/2_ spectra corresponding to Ni–OH and Co–OH, respectively (Fig. S4†). The O 1s level comprises a metal–oxygen bond in the MO_6_ octahedron of the hexagonal LDH lattice (529.2 eV), the –OH groups of LDH (529.8 eV) and defective O (531.2 eV) generated due to the presence of low valent Co^2+^ ions. The LDH nanosheets on the surface of Cu-Ws show a broad PXRD reflection at 23.86° from the (006) plane of NiCo-LDH (Fig. S5a†). The position of this peak and that of the extremely weak (110) reflection at 2*θ* = 53–66° expectedly vary upon changing the Ni : Co ratio (Fig. S5b, c and Table S1†). Energy dispersive X-ray (EDX) analyses from five or more locations of the electrode confirm a good match of the Ni : Co ratio with the precursor composition (Table S2†). With Ni : Co ratios of 1 : 1, 1 : 2 and 2 : 1, the lattice parameter *a* (=2*d*_110_) changes to 3.15, 2.98 and 2.86 Å and the lattice parameter *c* (=6*d*_006_) varies as 22.34, 23.13 and 23.33 Å, respectively.

**Fig. 2:**
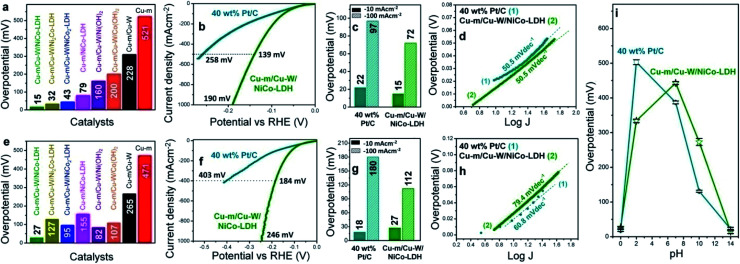
HER performance of Cu-m/Cu-W/NiCo-LDH and its comparison with the HER activity of 40 wt% Pt/C and other catalysts. (a) HER overpotentials required to reach −10 mA cm^−2^ in 1 M KOH for different catalysts. (b) HER polarization curves (iR-corrected) in 1 M KOH, (c) overpotentials required to reach −10 and −100 mA cm^−2^, (d) Tafel plots in 1 M KOH for Cu-m/Cu-W/NiCo-LDH and 40 wt% Pt/C. (e) HER overpotentials required to reach −10 mA cm^−2^ in 0.5 M H_2_SO_4_ for different catalysts. (f) HER polarization curves (iR-corrected) in 0.5 M H_2_SO_4_, (g) overpotentials required to reach −10 and −100 mA cm^−2^, (h) corresponding Tafel plots in 0.5 M H_2_SO_4_ and (i) required overpotential across the pH spectrum to reach −10 mA cm^−2^ for Cu-m/Cu-W/NiCo-LDH and 40 wt% Pt/C.

The electrochemical HER performance of the electrodes was first assessed in 1 M KOH at a scan rate of 10 mV s^−1^ in a typical three-electrode setup under a N_2_ atmosphere. The potential of the reference electrode was determined in a H_2_ saturated electrolyte and converted to the RHE in order to determine the exact scanning potential for the HER. In all the cases, the potential of the Ag/AgCl reference electrode remained unaltered after the electrochemical measurements under alkaline conditions (Fig. S6†). The best performing Cu-m/Cu-W/NiCo-LDH electrode (Ni : Co = 1 : 1) requires a minimal overpotential of 15 ± 6 mV to reach a current density of −10 mA cm^−2^ (Fig. 2a). It is indeed a rare occurrence wherein an electrode made solely of earth-abundant elements can match or surpass the electrocatalytic performance of 10 wt% Pt/C (Fig. S7†) and 40 wt% Pt/C (Fig. 2b and c). 40 wt% Pt/C needs 22 mV with the same mass loading and the relative trend is sustained at higher current densities. In contrast, the other catalysts and control electrodes such as Cu-m/Cu-W/Ni_2_Co-LDH, Cu-m/Cu-W/NiCo_2_-LDH, Cu-m/Cu-W/Ni(OH)_2_, Cu-m/Cu-W/Co(OH)_2_, Cu-W and bare Cu-m need an overpotential of 32, 43, 160, 200, 228 and 521 mV, respectively (Fig. 2a and S8a†). Moreover, the cathodic current density of the premier Cu-m/Cu-W/NiCo-LDH electrode increases rapidly and reaches −100, −500 and −1000 mA cm^−2^ at considerably low overpotentials of 72, 139, and 190 mV, respectively, suggesting its potential for widespread commercialization (Fig. 2b and c). The Tafel slope of Cu-m/Cu-W/NiCo-LDH (50.5 mV dec^−1^) although similar to that of 40 wt% Pt/C, is lower than that of the other electrodes suggesting Pt-like fast adsorption–desorption kinetics (Fig. 2d and S8b†), where the electrochemical desorption step is rate-determining according to the Volmer–Heyrovsky mechanism (Fig. S8c†).^44^ When measured at a slower scan rate of 5 mV s^−1^ to maintain the steady-state, the HER overpotential of Cu-m/Cu-W/NiCo-LDH remains the same and its Tafel slope gets better than 40 wt% Pt/C (Fig. S9†). At an even lower scan rate of 1 mV s^−1^, the electrode performance is comparable to that of platinum and surpasses that of 40 wt% Pt/C at higher current densities (Fig. S10a†). The best performance is obtained by 60 s electrodeposition of NiCo-LDH (Fig. S11a†) and 30 min chemical oxidation of Cu-m (Fig. S11b†). The HER activity remains unchanged on switching the counter electrode from Pt wire to graphite paper which substantiates the activity of this electrode to be intrinsic in nature and not due to any possible deposition of Pt on the working electrode (Fig. S11c†).

In 0.5 M H_2_SO_4_ too, Cu-m/Cu-W/NiCo-LDH displays the best HER activity among all other control electrodes (Fig. 2e and S8d†) and requires only a 27 ± 2 mV overpotential to achieve −10 mA cm^−2^ which is only 9 mV higher than the 18 mV overpotential for 40 wt% Pt/C (Fig. 2f and g) but is comparable with that of 10 wt% Pt/C (Fig. S7†). Interestingly this electrode rapidly surpasses the activity of Pt/C and achieves high current densities of −100 and −1000 mA cm^−2^ at a 112 and 246 mV overpotential, respectively (Fig. 2f and g). Either when iR-corrected or uncorrected in acidic and alkaline media or when measured at a lower scan rate of 1 mV s^−1^ in an acidic medium the activity of Cu-m/Cu-W/NiCo-LDH, elucidated from the LSV plots, remains comparable that of to 40 wt% Pt/C (Fig. S10†). At pH = 0, the Tafel slope of Cu-m/Cu-W/NiCo-LDH is 79.4 mV dec^−1^, lowest among the control samples in Fig. S6e† and comparable to that of Pt/C (60.8 mV dec^−1^) (Fig. 2h).

Cu-m/Cu-W/NiCo-LDH shows an excellent pH universality adjudged by the required overpotentials of 334, 443, and 273 mV at pH = 2, 7 and 10, to reach −10 mA cm^−2^, in comparison to 40 wt% Pt/C requiring 503, 388, and 132 mV, respectively (Fig. 2i, S12 and Table S3†). Other than pH = 0 and pH = 14, the activity is less proficient and sometimes inferior to that of 40 wt% Pt/C due to the low ionic conductivity of the solution at pH = 2, 7 and 10, and better electronic conductivity of platinum than NiCo-LDH. The mass activity is also comparable to that of the Pt/C electrode, as is observed by normalizing the catalytic current by catalyst loading (Fig. S13†). At a 30 mV overpotential, the turnover frequency (TOF) of the Cu-m/Cu-W/NiCo-LDH electrode is 0.3 and 0.32H_2_ s^−1^ in 1 M KOH and 0.5 M H_2_SO_4_, respectively advocating its high intrinsic activity (Discussion S1†). Rapid electron transfer is possible due to the least resistance to charge transfer kinetics (*R*_CT_) of this electrode both in 1 M KOH and 0.5 M H_2_SO_4_ (Fig. S14†). Likewise the double layer capacitance (*C*_dl_), proportional to the electrochemically active surface area (ECSA) and surface roughness factor (*R*_f_), is highest at 19.8 mF cm^−2^ when Ni : Co is 1 : 1 (Fig. S15†) implying the highest abundance of active sites.^45^

**Fig. 3:**
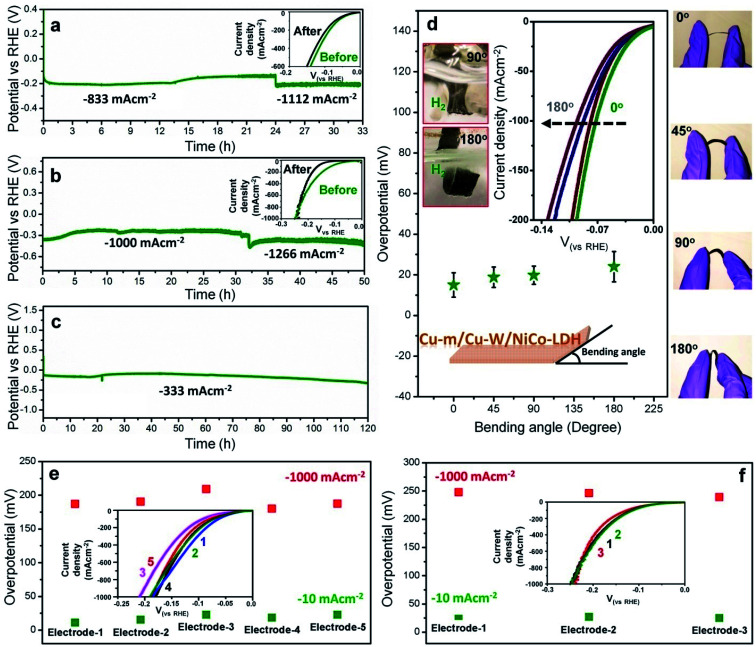
Stability and flexibility tests of the Cu-m/Cu-W/NiCo-LDH electrode. Chronopotentiometric stability test (a) for 33 h at −833 and −1112 mA cm^−2^ in 1 M KOH, (b) for 50 h at −1000 and −1266 mA cm^−2^ in 0.5 M H_2_SO_4_ and (c) for 120 h at −333 mA cm^−2^ in 1 M KOH. The insets in (a, b) show steady state LSV plots before and after the stability tests. (d) Flexibility studies showing the overpotentials required to reach −10 mA cm^−2^ in 1 M KOH at different bending angles of the current collector. The insets show the corresponding digital images. Reproducibility tests of the HER performance with different batches of Cu-m/Cu-W/NiCo-LDH electrodes in (e) 1 M KOH and (f) 0.5 M H_2_SO_4_.

The unparalleled self-supported Cu-m/Cu-W/NiCo-LDH electrode is extremely stable under operating conditions at both pH values from low to high negative current densities (Fig. 3a–c). Chronopotentiometric tests in 1 M KOH show unaltered stability both at −833 and −1112 mA cm^−2^ which favors rapid mass diffusion of the reactant and product (Fig. 3a), with no change in the linear sweep voltammetry (LSV) profile after 33 h (Fig. 3a inset). In 0.5 M H_2_SO_4_, except for a slight attenuation, the electrode has unprecedented durability at −1000 and −1266 mA cm^−2^ for at least 50 h (Fig. 3b and inset). A long term chronopotentiometric stability test for 120 h at −333 mA cm^−2^ (Fig. 3c) and 100 h at −1 A cm^−2^ (Fig. S16 and ESI Movie 1†) with vigorous release of hydrogen bubbles in 1 M KOH further attests to its suitability for commercial applications, besides 5000 cycle tests at both pH values and chronoamperometric evaluation for 24 h at only a 9 mV overpotential in 1 M KOH (Fig. S17†). The chronoamperometric stability at low overpotential strengthens the fact that the current is not contributed by any metal reduction or by *C*_dl_.

After the chronopotentiometric stability test at −1 A cm^−2^ at pH 14 (Fig. S16†), the crystal structure, composition and morphology of NiCo-LDH are well maintained (Fig. S18†). After the stability test at −1 A cm^−2^ at pH 0 (Fig. 3b), the 2D LDH sheets partly transform into ∼20 nm particles although the crystal integrity and composition are preserved (Fig. S18†). The electrodes carry the added advantage of being mechanically flexible and robust which ensures unperturbed mass diffusion for long hours of operation. As demonstrated in Fig. 3d, at different bending angles of the electrode, the required HER overpotential remains unaffected without any structural failure. Fig. 3d insets show smooth release of gas bubbles even under extreme mechanical stress. Almost all the charge is consumed for hydrogen production, validated by gas chromatography (GC) without any parasitic side reaction as confirmed by the eudiometric method at constant current densities, wherein the electrode shows power conversion efficiencies of 99.5% at −300 mA cm^−2^ in 1 M KOH and 94.9% at −667 mA cm^−2^ in 0.5 M H_2_SO_4_ (Fig. S19†). The HER activity is distinctly reproducible at both pH values across different batches of electrodes (Fig. 3e and f) and expectedly it is better or comparable to that of the best known catalysts reported to date (Tables S4 and S5†).^5,10–12,14–27,46–48^ When coupled to NiFe-LDH, overall water splitting is obtained at 1.508 V by the NiFe-LDH (+) || Cu-m/Cu-W/NiCo-LDH (−) electrolyzer to achieve a current density of 10 mA cm^−2^ compared to IrO_2_ (+) || 40 wt% Pt/C (−) which needs 1.55 V along with exceptional stability for almost 70 h at 333 mA cm^−2^, making it suitable for practical applications (Fig. S20†).

The exceptional catalytic performance of Cu-m/Cu-W/NiCo-LDH has much to do with the Cu-m/Cu-W substrate. The feat of surpassing the Pt-activity in catalyzing the HER is attributed to our strategy of growing Cu-W since the activity is lowered by NiCo-LDH electrodeposited only on Cu-m (Fig. 2a and S6a†), and a previously reported Cu-foam supported HER catalyst requiring a higher overpotential of 54 mV.^31^ The advantages of the woven like porous structure of the Cu-m/Cu-W substrate for improved mass transport and fast release of gas bubbles are more evident when NiCo-LDH is deposited on Cu-W grown from chemical oxidation of Cu foam or NiCo-LDH deposited directly on Ni foam, all of which show subservient activities (Fig. S21†). When NiCo-LDH is deposited on Ni foam, the LDH sheets surround the flakes on a rough Ni foam surface unlike the large spread of LDH 2D sheets over Cu-W (Fig. S22†). The versatility of the Cu-m/Cu-W substrate is apparent when different catalysts such as NiMo-catalysts, NiCr-LDH and NiMn-LDH deposited on Cu-m/Cu-W show improved HER performance than those deposited on the commonly used CFP (Fig. S23†). Replacing the Cu-m/Cu-W core with Ni nanowires grown on Ni foam, and electrodepositing NiCo-LDH also demonstrates promising HER activity (Fig. S24†), thus validating the universality of our approach. Nevertheless with the same substrate these Ni-containing catalysts lack the exceptional HER activity of NiCo-LDH, which has the synergism of Ni predominating in OH^−^ desorption and Co facilitating the Heyrovsky step.^41,42,49^ With NiCo-LDH on CFP, the activity is similarly weakened accompanied by a drop in ECSA (Fig. S25a–d†). Normalizing the catalytic current of Cu-m/Cu-W/NiCo-LDH and CFP/NiCo-LDH using their respective ECSA accentuates the significant role of the metallic Cu-W core (Fig. S25e†). The high density of electronic states near the Cu Fermi energy level (*E*_F_) necessary for sublime electron transport is discerned from the valence band XPS analyses (Fig. S26†). The electron density at *E*_F_ contributed by the electrically conducting Cu-W core remains unaffected post NiCo-LDH decoration, which helps in promoting unhindered electron transfer with minimal resistance.

**Fig. 4:**
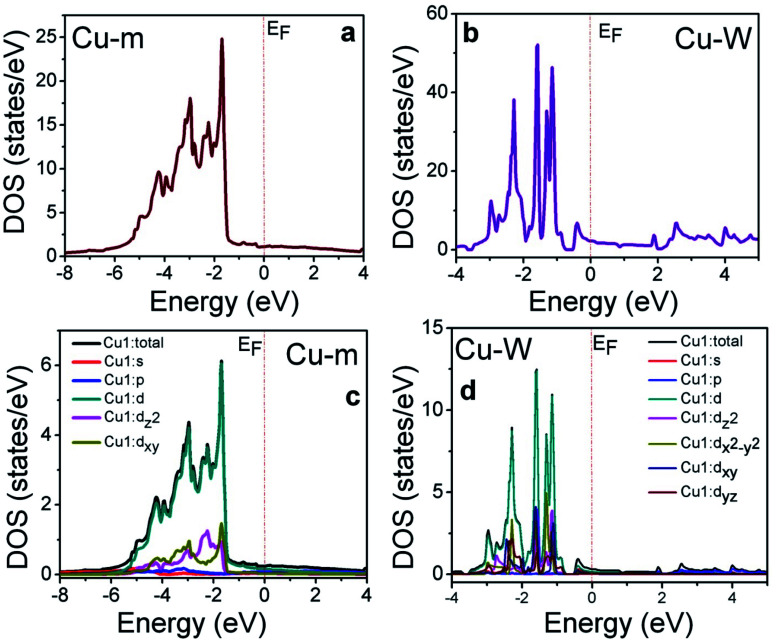
Results of electronic states calculations. Total DOS plots of (a) Cu-m and (b) Cu-W. PDOS plots the of Cu1 atom and different d-orbitals for (c) Cu-m and (d) Cu-W.

The benefits of introducing one-dimensional Cu-W on bulk-like Cu-m is better understood from density of states (DOS) calculations within the full potential linearized augmented plane wave (FP-LAPW) based on density functional theory (DFT) as implemented in the Wien2k code.^50^ The density of states (DOS) calculated from generalized gradient approximation (GGA),^51^ for both Cu-m from −8 eV to 4 eV and Cu-W from −4 to 5 eV is shown in Fig. 4, where *E*_F_ is represented by the dotted lines. In the case of Cu-W, the DOS above 3 eV in the conduction band side is due to the vacuum that was applied to discontinue the atomic wave function.

In both Cu-m and Cu-W, the major contribution comes from the position and contribution of the Cu-d band state. For Cu-m, the partial DOS (PDOS) of the Cu-d band (Fig. 4c) arises mainly from hybridization of d_*x*^2^–*y*^2^_ (similar DOS with d_*z*^2^_) and d_*xy*_ (similar DOS with d_*yz*_ and d_*yz*_) near *E*_F_ (−2 to 0 eV) where the orbitals in between the Cartesian axes (*i.e.*, d_*xy*_, d_*yz*_, d_*xz*_) dominate over those along the axes (d_*z*^2^_, d_*x*^2^–*y*^2^_). Due to the one-directional axis and orientation of the Cu-W structure, the PDOS of the Cu-d state and thereby the total DOS of Cu-W are visibly up shifted towards *E*_F_ as compared to Cu-m (Fig. 4c and d). However, since the total number of d electrons has to be conserved and the d-band cannot pass through *E*_F_, the total DOS and d-band shifting towards *E*_F_ are likely due to the contribution of t_2g_ and e_g_ orbital splitting. The d_*z*^2^_ and d_*x*^2^–*y*^2^_ orbitals (along the axis) are at higher density of states near to E_F_ and among those in between the axes, the DOS of the d_*xy*_ orbital is closer to *E*_F_ than that of d_*xz*_ and d_*yz*_. A higher DOS of the orbitals oriented along the Cartesian axes implies higher availability of charge carriers to be favorably transferred to execute the redox reactions which clearly manifests the better propensity of electron transfer from Cu-W to NiCo-LDH than that from Cu-m.

The free energy of adsorption on the catalyst supported on Cu-W could not be elucidated by DFT calculations due to the relatively amorphous nature of our NiCo-LDH catalyst. Recent calculations have shown that NiCo-LDH can efficiently dissociate the water molecules with a very low energy barrier,^52^ which makes LDH a lucrative HER electrocatalyst in alkaline media. The second step *i.e.* optimum proton adsorption governs the catalyst performance in acidic and alkaline media *via* H_2_ generation. Metallic structures such as Pt show Gibbs free energy for the adsorption of H* close to 0 eV.^53^ Similar to Pt, our self-supported electrode shows metallic character with high density of electronic states near *E*_F_ (Fig. S26†), which helps in optimal adsorption of protons after the water dissociation step in the alkaline medium and from the electrolyte in strong acids to generate hydrogen fuel. Combining the predicted water dissociation ability of NiCo-LDH and optimal H* adsorption energy of metallic nanostructures, our strategy yields an unprecedented self-supported framework that can efficiently dissociate water along with a decent proton adsorption ability to boost the HER in alkaline and acidic media.

To conclude we have demonstrated the design of a Cu-m/Cu-W/NiCo-LDH self-supported and flexible current collector that outperforms the noble metal based state-of-the-art Pt/C in catalyzing the electrochemical HER across the entire pH spectrum with high current density. This electrode solely comprising earth-abundant elements needs minimal overpotentials of 15 and 27 mV to provide a −10 mA cm^−2^ current density at pH 14 and pH 0, respectively, along with exceptional durability at high current densities. The mass activity, TOF and pH universality are found to be on a par with 40 wt% Pt/C. The key to the design of this flexible current collector is the growth of Cu-Ws on top of Cu-m, validated by several control experiments with different HER catalysts deposited both on Cu-Ws and Cu-m/Cu-Ws. The exceptional HER performance is in tune with the remarkably high density of electronic states near the *E*_F_ of Cu-Ws, which is necessary for sublime electron transfer to the electrodeposited NiCo-LDH catalyst. Besides the electrochemical HER, the concept of fabricating self-supported electrodes by wrapping electrically conducting nanowires with catalyst nanostructures is extremely promising for endless avenues for energy conversion and storage devices such as flexible and portable fuel cells, metal-ion and metal-air batteries, supercapacitors, photovoltaic devices and photoconductors.

## Experimental section

### Materials

Cu mesh (Alfa Aesar, 0.11 mm), cobalt(ii) sulphate heptahydrate (Co(SO_4_)_2_, 7H_2_O; Merck, ≥98%), nickel(ii) sulphate heptahydrate (Ni(SO_4_)_2_, 7H_2_O; Merck, ≥98%), potassium hydrogen carbonate (KHCO_3_; Merck, ≥99.5%), ammonium per-sulphate solution (Lobachemie, ≥98%) ammonia solution (Merck, ≥25%), nickel(ii) chloride hexahydrate (NiCl_2_·6H_2_O; Merck, ≥99%), chromium(iii) chloride hexahydrate (CrCl_3_·6H_2_O; Merck, ≥96%), manganese(ii) chloride tetrahydrate (MnCl_2_·4H_2_O; Merck, ≥96%), sodium hydroxide (NaOH; Merck, ≥97%), sodium dodecyl sulphate (SDS; Merck, ≥98%), potassium hydroxide pellets (KOH; Merck, ≥85%), ethanol (C_2_H_5_OH; Merck ≥99.9%), Toray carbon fiber paper (CFP; Alfa Aesar), commercial Pt/C (40 wt%, Merck), ammonium molybdate ((NH_4_)_6_Mo_7_O_24_; 97%, Merck), sodium chloride (NaCl; 99%, Merck), sodium hydrogen citrate (Na_2_C_6_H_6_O_7_; 98%, Merck), potassium hexachloroiridate (K_2_IrCl_6_; 99%, Sigma-Aldrich), nickel nitrate hexahydrate (Ni(NO_3_)_2_·6H_2_O; 98%, Merck), iron nitrate nonahydrate (Fe(NO_3_)_3_·9H_2_O; 98%, Merck) and Nafion perfluorinated resin solution (5 wt%, Merck) were used without further purification.

### Preparation of the Cu-m/Cu-W electrode

The Cu-m electrodes of a 2 × 0.5 cm^2^ area were cleaned successively with conc. HCl, deionized water and acetone by sonication for 20 min in each case followed by 2 min air drying. EDX analyses showed the absence of any possible impurity. To grow Cu(OH)_2_ nanowires on Cu-m by chemical oxidation, the cleaned electrodes were dipped in a 14 ml aqueous solution containing 0.84 g NaOH and 0.24 g (NH_4_)_2_S_2_O_8_.^54^ The time of chemical oxidation varied as 10, 30 and 60 min. The Cu(OH)_2_ nanowires were calcined at 180 °C for 1 h in air for phase transformation to CuO. Metallic Cu-Ws were prepared by electrochemical reduction of the CuO nanowires supported on Cu-m at −1.08 V in a 1 M KHCO_3_ electrolyte (pH = 7.9) in a three electrode configuration using Cu-m as the working electrode, Pt wire as the counter electrode and Ag/AgCl (3 M KCl) as the reference electrode in the potentiostatic method. The successful conversion of CuO nanowires to Cu-Ws was indicated by the appearance of H_2_ bubbles on the electrode surface. Finally the Cu-m/Cu-W working electrodes were removed followed by cleaning with distilled water and drying in air for 1 h.

### Preparation of Cu-m/Cu-W/Ni_*x*_Co_*y*_-LDH (*x* : *y* = 1 : 1, 1 : 2, and 2 : 1) electrodes

NiCo-LDH was electrochemically deposited on Cu-m/Cu-W electrodes by chronoamperometry at a fixed potential of −1.2 V in the required volume (*x* : *y*) ratios of two aqueous solutions: 0.4 M NH_4_Cl + 0.4 M CoSO_4_·7H_2_O and 0.4 M NH_4_Cl + 0.4 M NiSO_4_·7H_2_O. Electrodeposition was conducted in a three electrode configuration using Cu-m/Cu-W as the working electrode, Pt wire as the counter electrode and Ag/AgCl (3 M KCl) as the reference electrode. The LDH shell thickness was controlled by tuning the time of electrodeposition to 30, 60 and 90 s. The working electrodes were finally detached followed by cleaning with distilled water and drying in air for 1 h. All the experiments were performed under a N_2_ atmosphere.

### Preparation of the Cu-m/Cu-W/M(OH)_2_ (M = Ni, Co) electrode

For electrodeposition of only Ni(OH)_2_ or Co(OH)_2_ on the Cu-W electrode, the above procedure was followed but in solutions containing either NiSO_4_·7H_2_O or CoSO_4_·7H_2_O.

### Preparation of CFP/NiFe-LDH

CFP/NiFe-LDH was prepared by electrochemical deposition using CFP as the working electrode, Pt wire as the counter electrode and Ag/AgCl (3 M KCl) as the reference electrode in an aqueous electrolyte containing 3 mM Ni(NO_3_)_2_·6H_2_O and 3 mM Fe(NO_3_)_3_·9H_2_O. The electrodeposition was carried out at −1.6 V for 100 s in potentiostatic mode until the electrode surface turned yellowish accompanied by incessant generation of H_2_ bubbles. Finally, the working electrodes were removed followed by cleaning with distilled water and drying in air for 1 h.

### Preparation of IrO_2_

The synthesis protocol of IrO_2_ was adapted from a literature report.^55^ An aqueous solution was prepared by adding 50 ml K_2_IrCl_6_ (0.2 mmol) to another aqueous solution of 0.16 g C_6_H_5_Na_3_O_7_·2H_2_O (0.63 mmol). The solution pH was adjusted to 7.5 by repeatedly adding a 0.25 M NaOH solution at room temperature followed by repeated heating under stirring at 95 °C for 30 min. The centrifuged and dried product was finally calcined in air at 400 °C for 30 min.

### Preparation of Cu-m/Cu-W/NiMo-catalyst and CFP/NiMo-catalyst electrodes

A bimetallic NiMo catalyst was electrodeposited onto Cu-m/Cu-W or CFP substrates in a galvanostatic mode from an electrolytic bath comprising Ni(SO_4_)_2_ (0.017 M), Na_3_C_6_H_5_O_7_ (0.016 M), (NH_4_)_6_Mo_7_O_24_ (0.36 mM) and NaCl (0.28 M).^7^ A 27 wt% aqueous NH_3_ solution was added to adjust the pH to 9.5. The electrodeposition was performed in an electrochemical cell with a CFP or Cu-m/Cu-W working electrode, Pt wire counter electrode and Ag/AgCl (3 M KCl) reference electrode at a constant cathodic current density of −100 mA cm^−2^ for 3600 s. A uniform dark-gray film coated on the surface of the working electrode was obtained with simultaneous generation of bubbles. The electrodes were washed multiple times with DI water and absolute ethanol before drying at 60 °C for 30 min. The final catalyst loading was determined to be 4 mg cm^−2^ by weighing the dried electrode.

### Preparation of Cu-m/Cu-W/NiMn-LDH and CFP/NiMn-LDH electrodes

NiMn-LDH was prepared by a similar electrochemical deposition method in an aqueous electrolyte containing equal volumes of 0.04 M NiCl_2_·6H_2_O and 0.04 M MnCl_2_·4H_2_O in addition to 0.04 M NH_4_Cl. Electrodeposition was carried out at −1.2 V for 60 s in potentiostatic mode until the electrode surface turned black with incessant generation of H_2_ bubbles. Finally, the working electrode was cleaned with distilled water and dried in air for 1 h.

### Preparation of Cu-m/Cu-W/NiCr-LDH and CFP/NiCr-LDH electrodes

The above electrodeposition procedure was followed but with a different electrolyte containing equal volumes of 0.04 M CrCl_3_·6H_2_O and 0.04 mM NiCl_2_·6H_2_O along with 0.04 M NH_4_Cl. The electrodeposition was carried out at −1.5 V for 360 s. The working electrode was similarly obtained by cleaning and drying in air for 1 h.

### Preparation of Ni-foam/Ni–W/NiCo-LDH electrodes

Nickel hydroxide nanowires were synthesized on top of Ni foam according to a literature report.^56^ Briefly, nickel foam was first cleaned with an acid, deionized water and acetone followed by introducing it into a 4 mol L^−1^ NaOH solution and heating at 60 °C for 12 h under sealed conditions. The product was washed with deionized water. After drying, the electrode was reduced to obtain metallic Ni followed by electrodeposition of NiCo-LDH for 60 s. The electrode was washed and dried for further use.

### Physical characterization

A Rigaku (mini flex II, Japan) powder X-ray diffractometer with Cu Kα radiation was used for PXRD measurements. While a Carl Zeiss SUPRA 55VP was used for recording the FESEM images, an Oxford Instruments X-Max system with INCA software was used for the EDX spectra. TEM images were recorded with an isopropanol dispersion of the samples drop-cast on carbon coated Cu-based grids (Ted Pella, Inc., 300 mesh Cu) using a JEOL, JEM-2100F microscope with a 200 kV electron source at the DST-FIST facility, IISER Kolkata. The TEM image of Cu-m/Cu-Ws was recorded directly with a sliced substrate. XPS studies were carried out by mounting the samples on copper stubs with silver paste in a commercial photoelectron spectrometer PHI 5000 Versa ProbeII, FEI Inc using an Al Kα (1486.6 eV) excitation source. The acquired data were background-corrected by the Shirley method, and the peaks were fitted using Fityk software, with Voigt peaks having 80% Gaussian and 20% Lorentzian components. All the data were corrected with respect to C 1s spectra. GC measurements were conducted using an Agilent 7890B (G3440B) instrument, IACS Kolkata.

### Electrochemical measurements

All electrochemical tests were carried out using a Biologic VSP-300 electrochemical workstation. The electrochemical HER performance and *C*_dl_ were determined in a conventional three electrode electrochemical cell and the overall water splitting test was performed in a two-electrode system in 1 M KOH. Cu-m/Cu-Ws or CFP was used as the working electrode, and platinum wire and Ag/AgCl (3 M KCl) served as the counter and reference electrodes, respectively. Graphite rod was also used as the counter electrode. CFP/IrO_2_ electrodes were prepared by dispersing 4 mg of the catalyst into 500 μl ethanol containing a 20 μl 5% Nafion solution and sonicated for 60 min. 100 μl of this catalyst ink was drop-cast on a 0.5 × 0.6 cm^2^ area of CFP and left to dry in air. The catalyst loading of CFP/40 wt% Pt/C was maintained the same as that of NiCo-LDH on Cu-m/Cu-W electrodes. The best performing electrode prepared with a 60 s deposition time has a loading of 1 mg cm^−2^ calculated from the weight difference of the Cu-m/Cu-W electrode before and after electrodeposition of NiCo-LDH. The current density was calculated considering the working surface area from a single side of the electrodes. All the potentials in the three-electrode measurements were 85% iR-corrected to compensate the effect of solution resistance unless specified and calibrated to the reversible hydrogen electrode (RHE). Before recording the electrochemical activity, all the working electrodes were saturated by 20 cyclic voltammetry (CV) scans at a scan rate of 200 mV s^−1^. LSV measurements were conducted at 10 mV s^−1^ in order to minimize the capacitive current and to maintain steady-state behavior. EIS studies were performed between 1 MHz and 10 mHz at a fixed potential of −1.15 V and −0.4 V *vs.* Ag/AgCl in 1 M KOH and 0.5 M H_2_SO_4_, respectively, to compare *R*_CT_ in the faradaic region since *R*_CT_ is inversely proportional to the rate of charge transfer. Faradaic efficiency was measured by using the eudiometric method in an air-tight vessel. *C*_dl_ was obtained by collecting CV scans at 10, 15, 20, 25 and 30 mV s^−1^ in the non-faradaic region (0 to 0.1 V *vs.* Ag/AgCl).^57^ GC measurements were conducted at a potential of −1.8 V *vs.* Ag/AgCl in 1 M KOH in the typical 3 electrode configuration.

### DFT calculations

The electronic structure of bulk Cu-m and one-dimensional Cu-Ws was investigated theoretically based on DFT formalism in the Wien2k code and GGA was used to adapt the electron exchange correlation. The cut-off parameter was *R*_MT_ × *K*_max_ = 7, where *K*_max_ represents the maximum value of the reciprocal lattice vector in the plane wave expansion and *R*_MT_ is the smallest atomic sphere radii of all atomic spheres. Cu-m has the space group *Fm*3̄*m* and Wyckoff positions (0,0,0). Cu-Ws were formed by the supercell method where the desired Cu–Cu distance was maintained at 10 Å along the *x*- and *y*-axis for discontinuing the atomic wave function in order to break the crystal symmetry along the *x* and *y* directions.^58^ The optimized lattice parameter for bulk Cu-m is 3.593 Å whereas in Cu-W, a *P* cell was created with a small *c* (=3.593 Å) and comparatively larger *a* (=*b*). The number of *k*-points for the bulk structure of Cu-m used in the irreducible part of the Brillouin zone was 3000. In the case of Cu-m, 343 *k*-points were generated with a division of 14 × 14 × 14 and for Cu-Ws, 225 *k*-points were generated with grid dimensions 7 × 7 × 18. The convergences of the total energy for both the structures in the self-consistent DFT calculations were maintained at 0.00001 Ry.

## Conflicts of interest

There are no conflicts of interest to declare.

## Supplementary Material

SC-011-D0SC00754D-s001

SC-011-D0SC00754D-s002

## References

[cit1] Cabán-Acevedo M., Stone M., Schmidt J., Thomas J., Ding Q., Chang H., Tsai M., He J., Jin S. (2015). Nat. Mater..

[cit2] Yu F., Zhou H., Huang Y., Sun J., Qin F., Bao J., Goddard W., Chen S., Ren Z. (2018). Nat. Commun..

[cit3] Halder G., Ghosh A., Parvin S., Bhattacharyya S. (2019). Chem. Mater..

[cit4] Kumar A., Bhattacharyya S. (2017). ACS Appl. Mater. Interfaces.

[cit5] Mahmood J., Li F., Jung S., Okyay M., Ahmad I., Kim S., Park N., Jeong H., Baek J. (2017). Nat. Nanotechnol..

[cit6] Kumar A., Chaudhary D. K., Parvin S., Bhattacharyya S. (2018). J. Mater. Chem. A.

[cit7] Sahasrabudhe A., Dixit H., Majee R., Bhattacharyya S. (2018). Nat. Commun..

[cit8] Jaramillo T., Jorgensen K., Bonde J., Nielsen J., Horch S., Chorkendorff I. (2007). Science.

[cit9] Lin H., Shi Z., He S., Yu X., Wang S., Gao Q., Tang Y. (2016). Chem. Sci..

[cit10] Mishra I., Zhou H., Sun J., Qin F., Dahal K., Bao J., Chen S., Ren Z. (2018). Energy Environ. Sci..

[cit11] Feng J., Tong S., Tong Y., Li G. (2018). J. Am. Chem. Soc..

[cit12] Gong M., Zhou W., Tsai M., Zhou J., Guan M., Lin M., Zhang B., Hu Y., Wang D., Yang J., Pennycook S., Hwang B., Dai H. (2014). Nat. Commun..

[cit13] Tabassum H., Guo W., Meng W., Mahmood A., Zhao R., Wang Q., Zou R. (2017). Adv. Energy Mater..

[cit14] Liu M., Zhang R., Zhang L., Liu D., Hao S., Du G., Asiri A., Kong R., Sun X. (2017). Inorg. Chem. Front..

[cit15] Wang W., Yang L., Qu F., Liu Z., Du G., Asiri A., Yao Y., Chen L., Sun X. (2017). J. Mater. Chem. A.

[cit16] Liu T., Liu D., Qu F., Wang D., Zhang L., Ge R., Hao S., Ma Y., Du G., Asiri A., Chen L., Sun X. (2017). Adv. Energy Mater..

[cit17] Zhang L., Lang Z., Wang Y., Tan H., Zang H., Kang Z., Li Y. (2019). Energy Environ. Sci..

[cit18] Yu J., Guo Y., She S., Miao S., Ni M., Zhou W., Liu M., Shao Z. (2018). Adv. Mater..

[cit19] Su J., Yang Y., Xia G., Chen J., Jiang P., Chen Q. (2017). Nat. Commun..

[cit20] Mao J., He C., Pei J., Chen W., He D., He Y., Zhuang Z., Chen C., Peng Q., Wang D., Li Y. (2018). Nat. Commun..

[cit21] Liu Y., Li X., Zhang Q., Li W., Xie Y., Liu H., Shang L., Liu Z., Chen Z., Gu L., Tang Z., Zhang T., Lu S. (2020). Angew. Chem., Int. Ed..

[cit22] Mahmood J., Anjum M., Shin S., Ahmad I., Noh H., Kim S., Jeong H., Lee J. S., Baek J. B. (2018). Adv. Mater..

[cit23] Peng Z., Wang H., Zhou L., Wang Y., Gao J., Liu G., Redfern S., Feng X., Lu S., Li B., Liu Z. (2019). J. Mater. Chem. A.

[cit24] Ding R., Chen Q., Luo Q., Zhou L., Wang Y., Zhang Y., Fan G. (2020). Green Chem..

[cit25] Li K., Li Y., Wang Y., Ge J., Liu C., Xing W. (2018). Energy Environ. Sci..

[cit26] Zhang J., Zhao Y., Guo X., Chen C., Dong C., Liu R., Han C., Li Y., Gogotsi Y., Wang G. (2018). Nat. Catal..

[cit27] Li F., Han G., Noh H., Jeon J., Ahmad I., Chen S., Yang C., Bu Y., Fu Z., Lu Y. (2019). Nat. Commun..

[cit28] Datta A., Kapri S., Bhattacharyya S. (2016). J. Mater. Chem. A.

[cit29] Al Cheikh J., Villagra A., Ranjbari A., Pradon A., Antuch M., Dragoe D., Millet P., Assaud L. (2019). Appl. Catal., B..

[cit30] Zhang L., Wang T., Sun L., Sun Y., Hu T., Xu K., Ma F. (2017). J. Mater. Chem. A.

[cit31] Wang W., Ren X., Hao S., Liu Z., Xie F., Yao Y., Asiri A., Chen L., Sun X. (2017). Chem.–Eur. J..

[cit32] Wang T., Zhang X., Zhu X., Liu Q., Lu S., Asiri A., Luo Y., Sun X. (2020). Nanoscale.

[cit33] Yu F., Yao H., Wang B., Zhang K., Zhang Z., Xie L., Hao J., Mao B., Shen H., Shi W. (2018). Dalton Trans..

[cit34] Ma X., Chang Y., Zhang Z., Tang J. (2018). J. Mater. Chem. A.

[cit35] Zhou C., Xia X., Wang Y., Zhong Y., Yao Z., Wang X., Tu J. (2017). J. Mater. Chem. A.

[cit36] Kang T. (2014). Appl. Phys. Lett..

[cit37] Feng J., Xu H., Dong Y., Ye S., Tong Y., Li G. (2016). Angew. Chem..

[cit38] Sivanantham A., Ganesan P., Shanmugam S. (2016). Adv. Funct. Mater..

[cit39] Yu L., Zhou H., Sun J., Qin F., Yu F., Bao J., Yu Y., Chen S., Ren Z. (2017). Energy Environ. Sci..

[cit40] Yu L., Zhou H., Sun J., Qin F., Luo D., Xie L., Yu F., Bao J., Li Y., Yu Y. (2017). Nano Energy.

[cit41] Quaino P., Juarez F., Santos E., Schmickler W. (2014). Beilstein J. Nanotechnol..

[cit42] Lai J., Huang B., Chao Y., Chen X., Guo S. (2019). Adv. Mater..

[cit43] Liu P., Yang S., Zhang B., Yang H. (2016). ACS Appl. Mater. Interfaces.

[cit44] Chen Z., Cummins D., Reinecke B., Clark E., Sunkara M., Jaramillo T. (2011). Nano Lett..

[cit45] Majee R., Chakraborty S., Salunke H. G., Bhattacharyya S. (2018). ACS Appl. Energy Mater..

[cit46] Cao Z., Chen Q., Zhang J., Li H., Jiang Y., Shen S., Fu G., Lu B., Xie Z., Zheng L. (2017). Nat. Commun..

[cit47] Pu Z., Amiinu I., Kou Z., Li W., Mu S. (2017). Angew. Chem., Int. Ed..

[cit48] Majee R., Kumar A., Das T., Chakraborty S., Bhattacharyya S. (2019). Angew. Chem., Int. Ed..

[cit49] Mahmood N., Yao Y., Zhang J., Pan L., Zhang X., Zou J. (2018). Adv. Sci..

[cit50] Blaha P., Schwarz K., Madsen G. K. H., Kvasnicka D., Luitz J., Laskowski R., Tran F. and Marks L. D., WIEN2k, An Augmented Plane Wave + Local Orbitals Program for Calculating Crystal Properties, ed. K. Schwarz, Techn. Universitat Wien, Austria, 2001

[cit51] Perdew J. P., Burke K., Ernzerhof M. (1996). Phys. Rev. Lett..

[cit52] Hu J., Zhang C., Jiang L., Lin H., An Y., Zhou D., Leung M., Yang S. (2017). Joule.

[cit53] Lukowski M., Daniel A., Meng F., Forticaux A., Li L., Jin S. (2013). J. Am. Chem. Soc..

[cit54] Zhang Y., Ma Y., Chen Y., Zhao L., Huang L., Luo H., Jiang W., Zhang X., Niu S., Gao D., Bi J., Fan G., Hu J. (2017). ACS Appl. Mater. Interfaces.

[cit55] Morris N. D., Mallouk T. E. (2002). J. Am. Chem. Soc..

[cit56] Xiao Q., Wang X., Huang S. (2017). Mater. Lett..

[cit57] Debnath B., Kumar A., Salunke H. G., Bhattacharyya S. (2017). J. Phys. Chem. C.

[cit58] Gao N., Li J. C., Jiang Q. A. (2013). Appl. Phys. Lett..

