# Supplementary material for: An earth-abundant bimetallic catalyst coated metallic nanowire grown electrode with platinum-like pH-universal hydrogen evolution activity at high current density
Source: Chem Sci. 2020 Mar 24;11(15):3893–902. doi: 10.1039/d0sc00754d (PMC8152688; doi:10.1039/d0sc00754d)
Supplement: SC-011-D0SC00754D-s001 [file SC-011-D0SC00754D-s001.pdf]

Electronic Supplementary Material (ESI) for Chemical Science.
This journal is © The Royal Society of Chemistry 2020

Supplementary Information

Earth-Abundant Bimetallic Catalyst Coated Metallic Nanowire Grown Electrode with Platinum-Like pH-Universal Hydrogen Evolution Activity at High Current Density

Sahanaz Parvin[#], Ashwani Kumar[#], Anima Ghosh and Sayan Bhattacharyya*

Department of Chemical Sciences, and Centre for Advanced Functional Materials, Indian Institute of Science Education and Research (IISER) Kolkata, Mohanpur -741246, India

# Equal contribution
*Email of correspondence: sayanb@iiserkol.ac.in

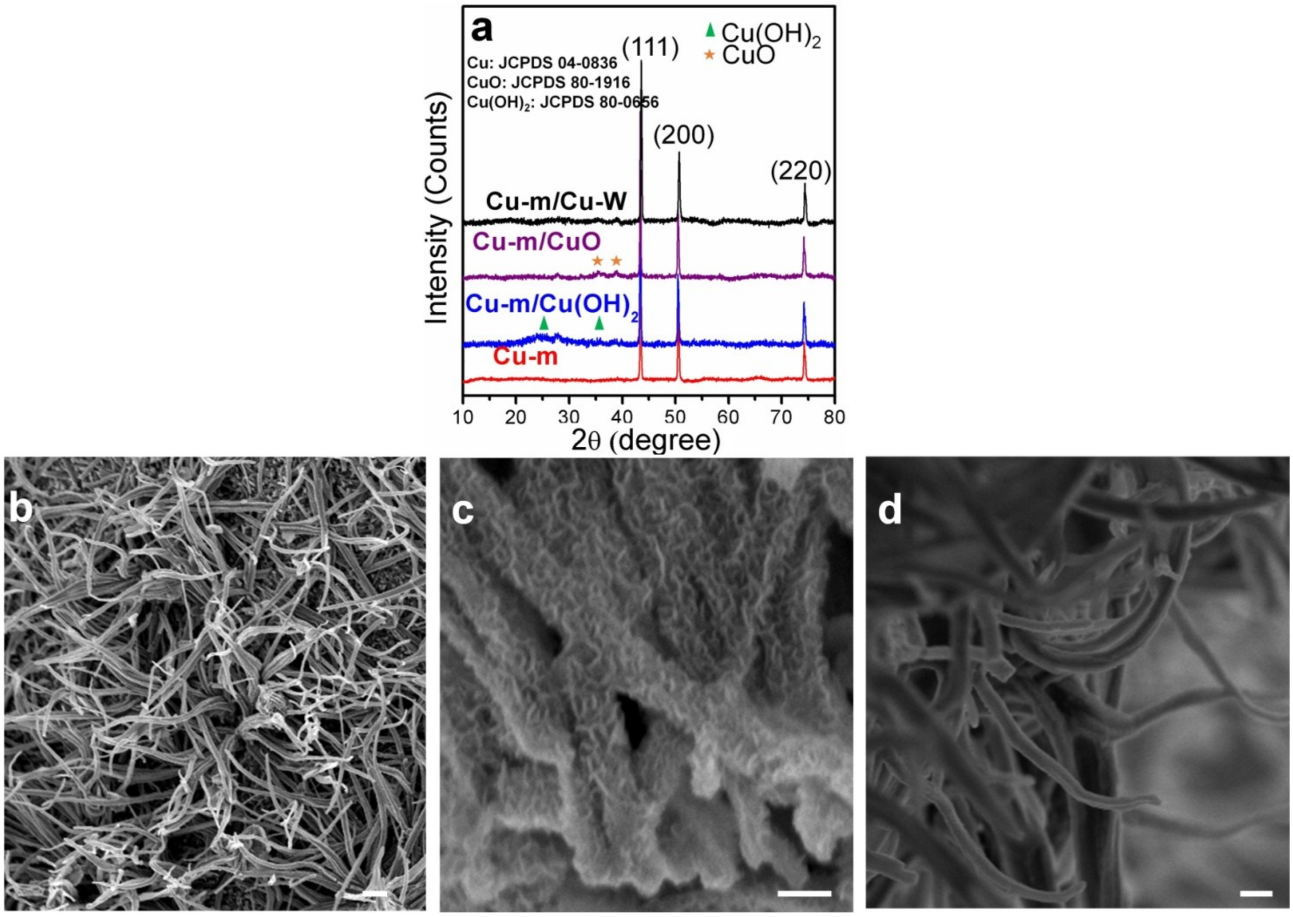

Figure S1. Structural and morphological evolution of the electrodes during the fabrication process. (a) PXRD patterns of Cu-m, Cu-m/$Cu(OH)_2$, Cu-m/CuO and Cu-m/Cu-W. FESEM images of (b) Cu-m/Cu-W (scale bar 1 μm) and (c) Cu-m/Cu-W/NiCo-LDH (scale bar 200 nm). (d) Cross-sectional FESEM image of Cu-m/Cu-W/NiCo-LDH (scale bar 300 nm).

The reflections of $Cu(OH)_2$ are according to JCPDS no. 80-056 and that of base-centered monoclinic CuO matches with JCPDS no. 80-1916. Arguably, among various x:y compositions of Ni_xCo_y-LDH, the catalyst with Ni:Co = 1:1 has the largest lattice parameter a suggesting less steric crowding with more exposure of active sites whereas the least c implies smallest interlayer distance between LDH sheets leading to higher electrical conductivity.

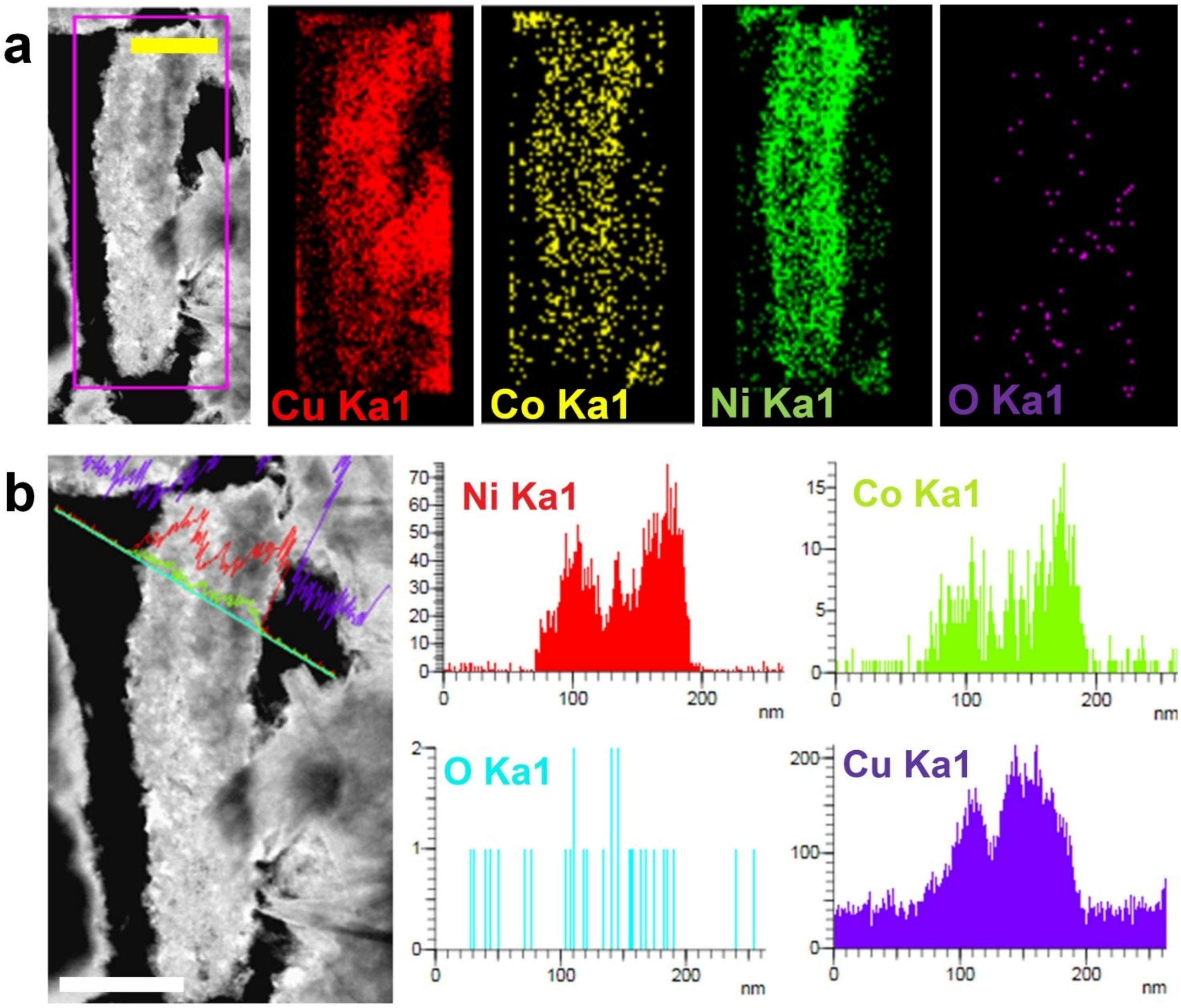

Figure S2. Microscopic elemental analyses of Cu-m/Cu-W/NiCo-LDH electrode. (a) HAADF-STEM mapping, and (b) elemental line scan (scale bars 100 nm).

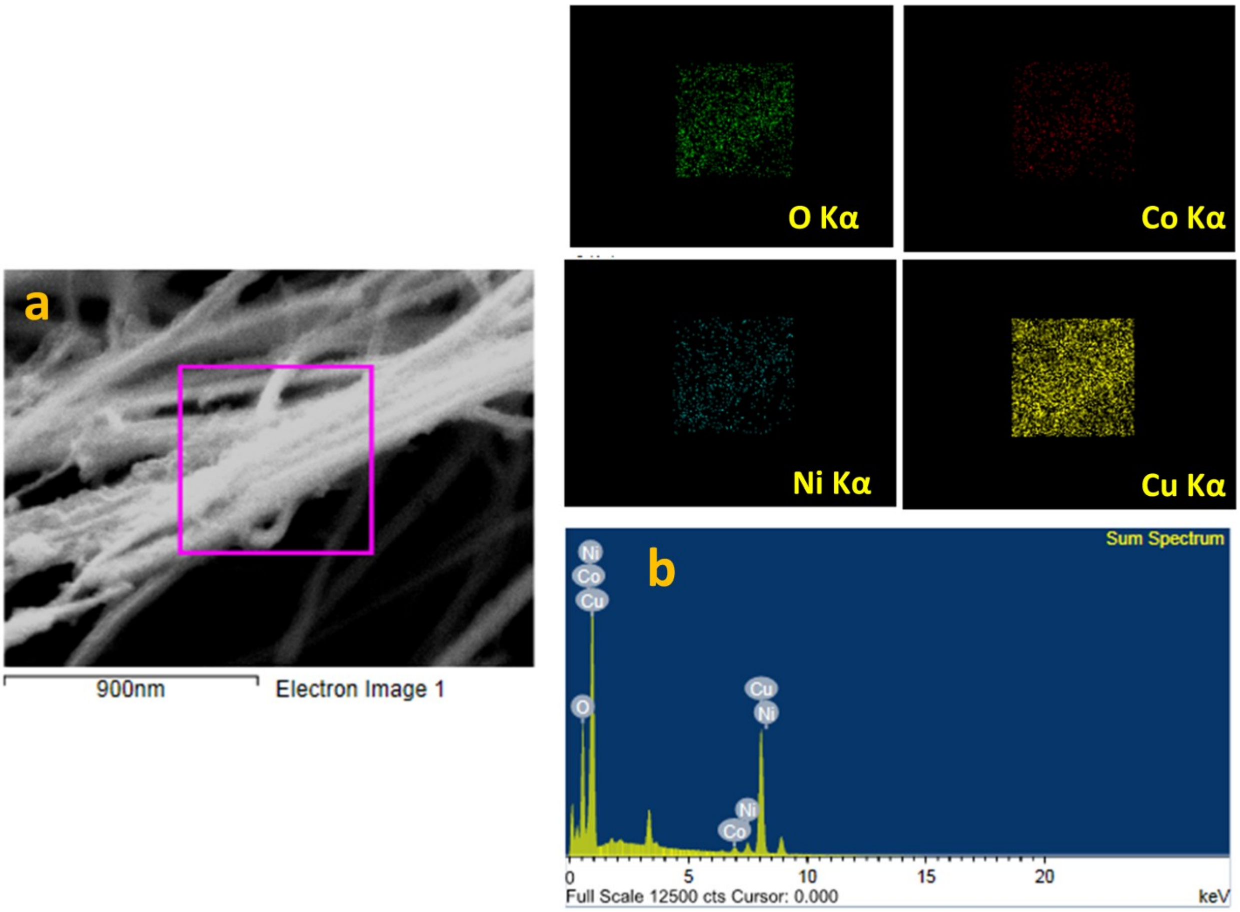

Figure S3. (a) EDS elemental mapping on the FESEM image of Cu-m/Cu-W/NiCo-LDH electrode and (b) the corresponding EDS spectrum.

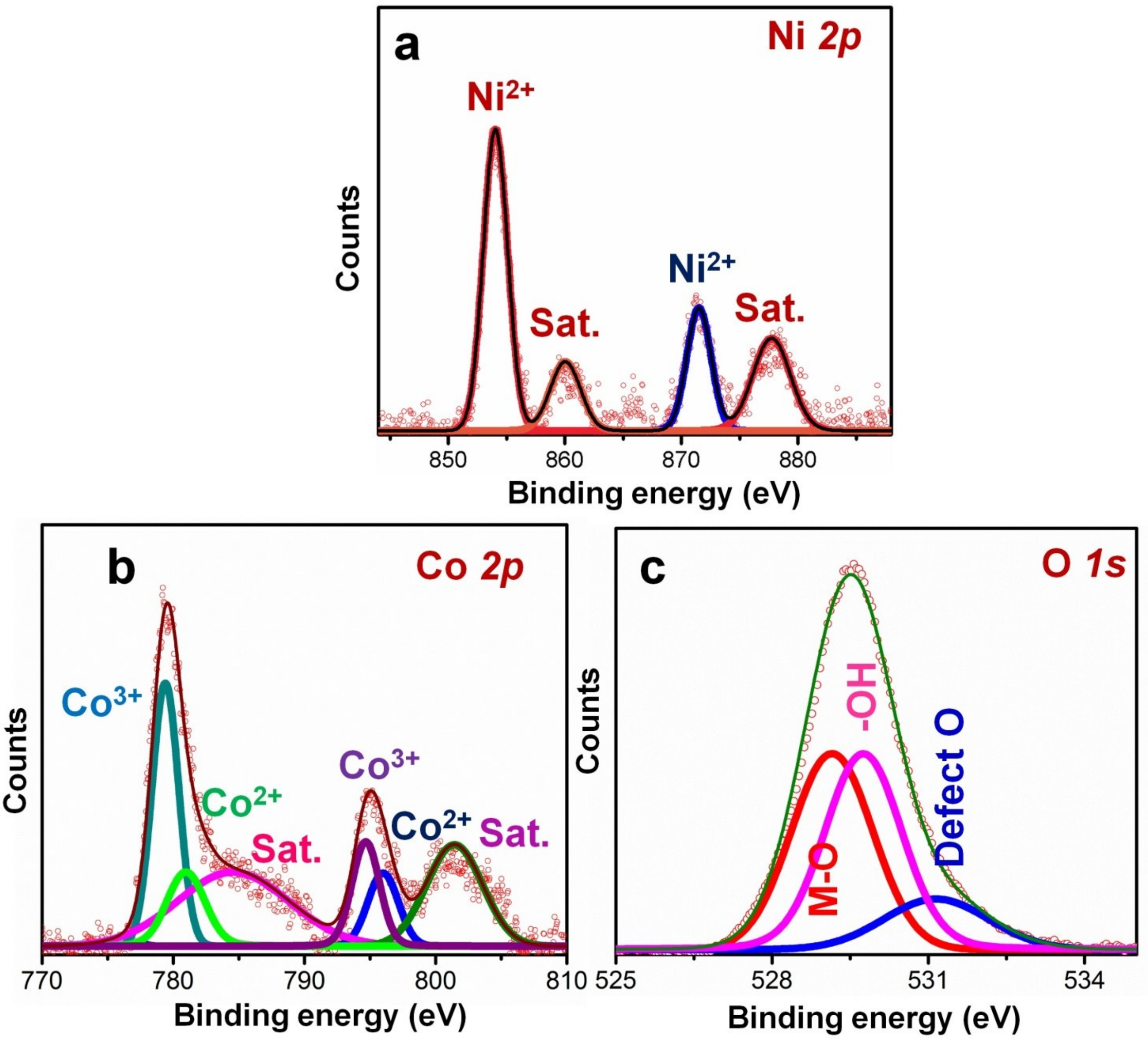

Figure S4. XPS spectra of Cu-m/Cu-W/NiCo-LDH electrode. Fitted deconvoluted XPS spectra of (a) Ni *2p*, (b) Co *2p* and (c) O *1s* levels. Open circles represent the experimental data and best fits are indicated by solid lines. Sat. denotes the satellite peaks.

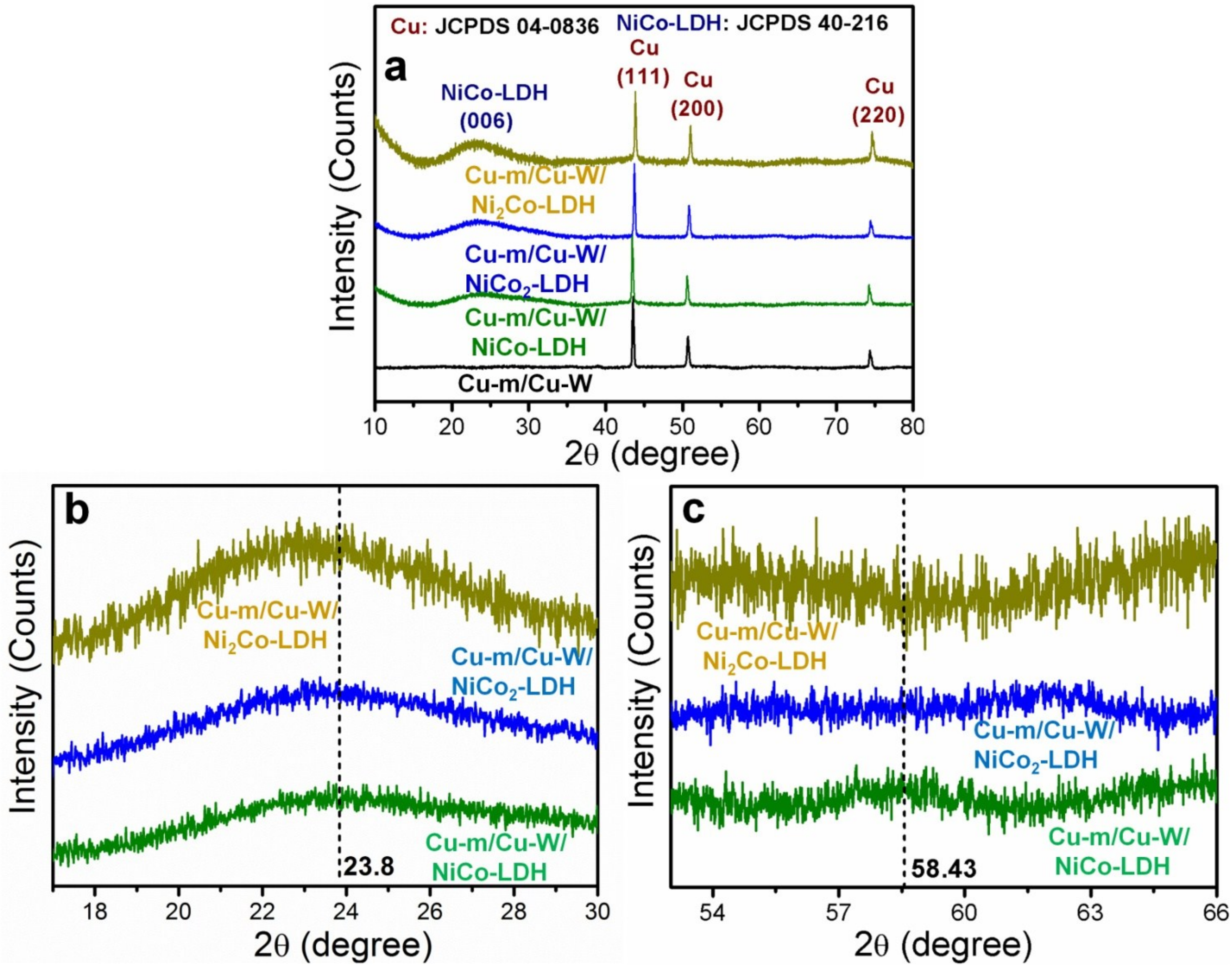

Figure S5. PXRD patterns of Cu-m/Cu-W/Ni_xCo_y-LDH electrodes (x:y = 2:1, 1:1, 1:2). PXRD patterns of the electrode (a) in the 2θ range 10-80°, and zoomed regions between (b) 2θ = 17-30°, and (c) 2θ = 53-66°.

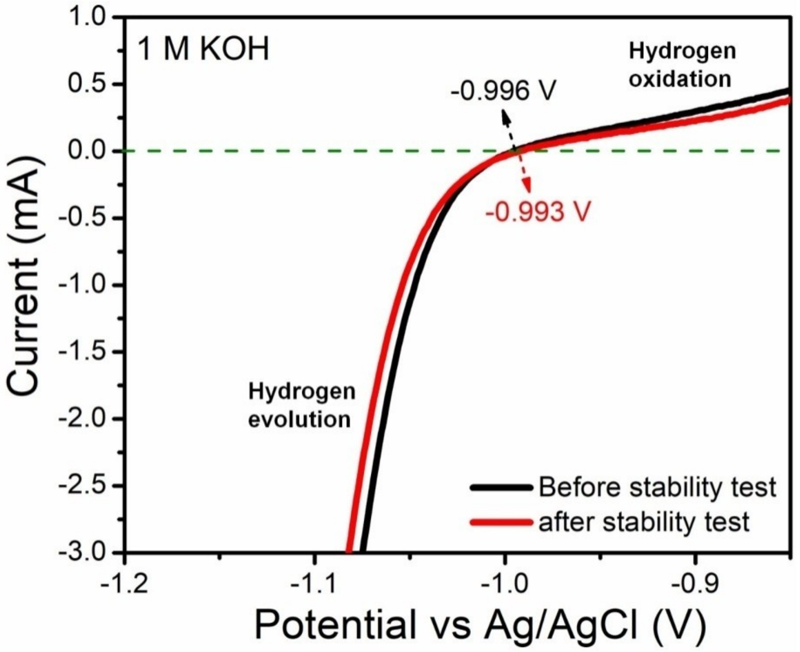

Figure S6. Calibration of Ag/AgCl reference electrode in H_2 saturated 1M KOH and conversion to RHE before and after electrocatalytic durability measurement.

The calibration of Ag/AgCl electrodes was performed in a standard three-electrode system with Pt mesh as both the working and counter electrodes. Ag/AgCl (3M KCl) electrode is used as the reference electrode. 1M KOH electrolyte was purged and saturated with high purity H_2 for 30 min. Linear sweep voltammograms (LSV) were recorded at a scan rate 5 mVs^{-1}. The potential at which the current crosses zero is the thermodynamic potential for hydrogen evolution/oxidation. Before performing the stability test, the zero current point is at -0.996 V $\approx$ -1 V, and therefore $E_{RHE} = E_{Ag/AgCl} + 1$ V. After the stability test with the same reference electrode, the zero current point remains at -0.993 V $\approx$ -1 V. This calibration data shows that the potential of Ag/AgCl (3M KCl) reference electrode remains unaltered in alkaline medium, which suggests the same potential window for HER. Hence the LSV curve for HER was recorded in the potential window of -1 to -1.8 V versus Ag/AgCl reference electrode which is equivalent to 0 to -0.8 V versus RHE in 1M KOH.

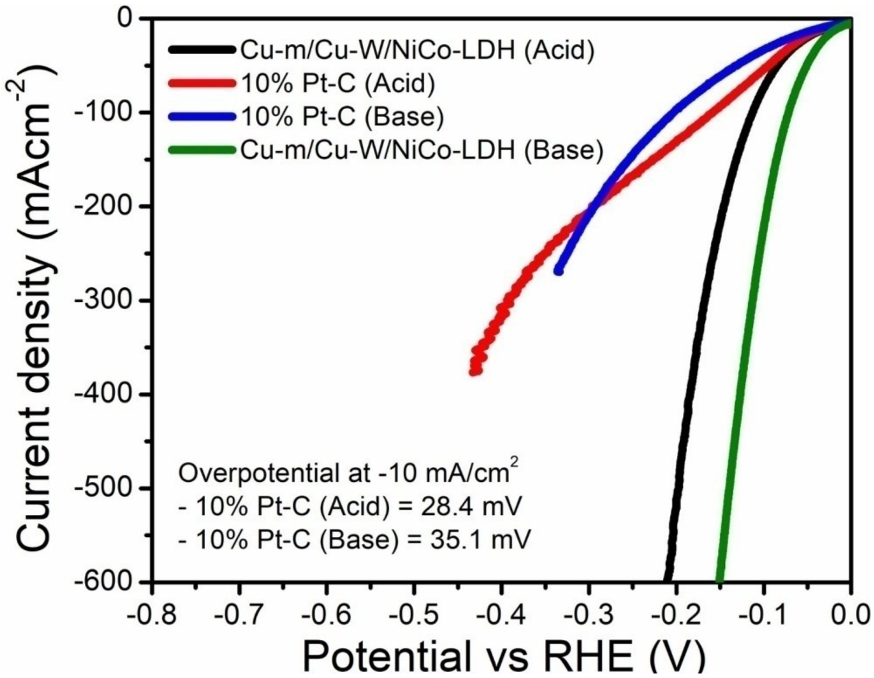

Figure S7. HER performance of Cu-m/Cu-W/NiCo-LDH and 10 wt% Pt/C in alkaline and acidic media.

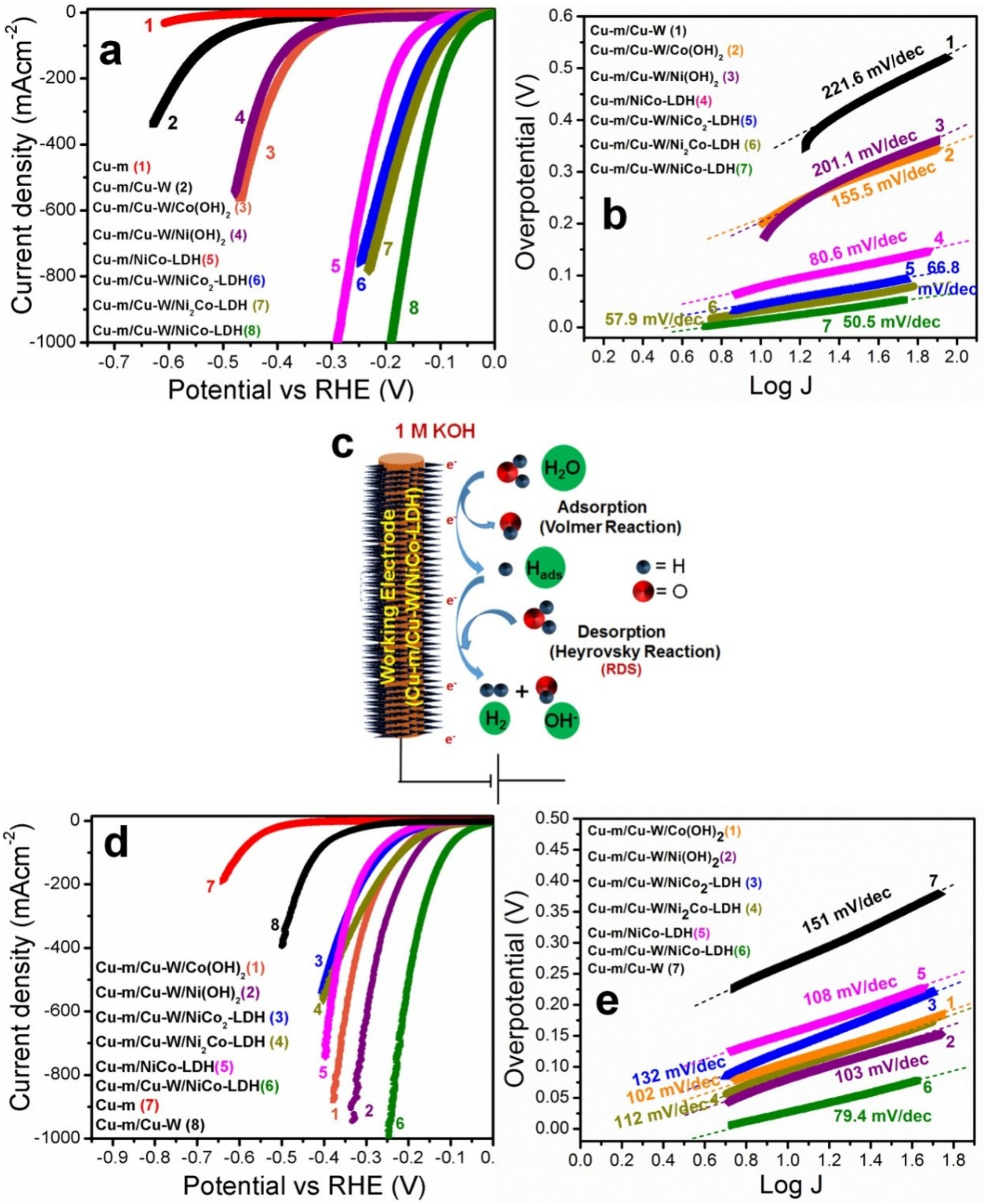

Figure S8. HER performance of different electrodes in alkaline and acidic media. (a) LSV, and (b) Tafel plots in 1M KOH. (c) Schematic of the Volmer-Heyrovsky mechanism. (d) LSV, and (e) Tafel plots in 0.5M H_2SO_4.

In the Volmer-Heyrovsky mechanism, when water molecules approach the electrical double layer surface of the catalyst, the H-O-H bond of water gets weakened subsequently followed by H-adsorption on the metal site and desorption of molecular H_2. Ni sites are more active in expediting the dissociation of H_2O (Volmer step) while Co sites facilitate the desorption of H_2 (Heyrovsky step).

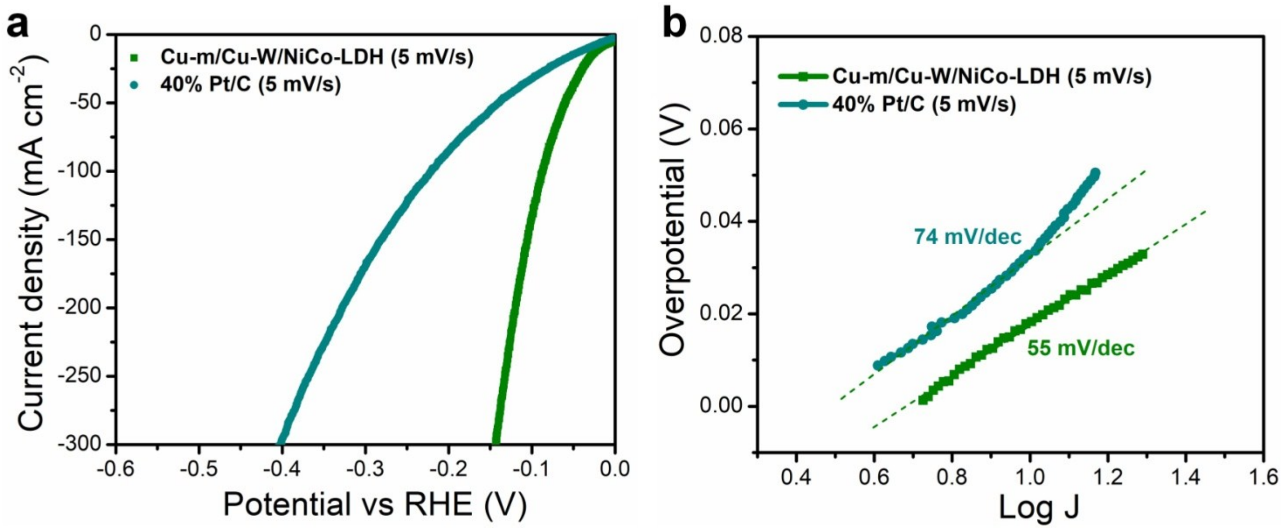

Figure S9. HER performance of Cu-m/Cu-W/NiCo-LDH and 40 wt% Pt/C with a slow scan rate of 5 mV/s. (a) LSV polarization plots of Cu-m/Cu-W/NiCo-LDH and 40 wt% Pt/C, and (b) corresponding Tafel plots.

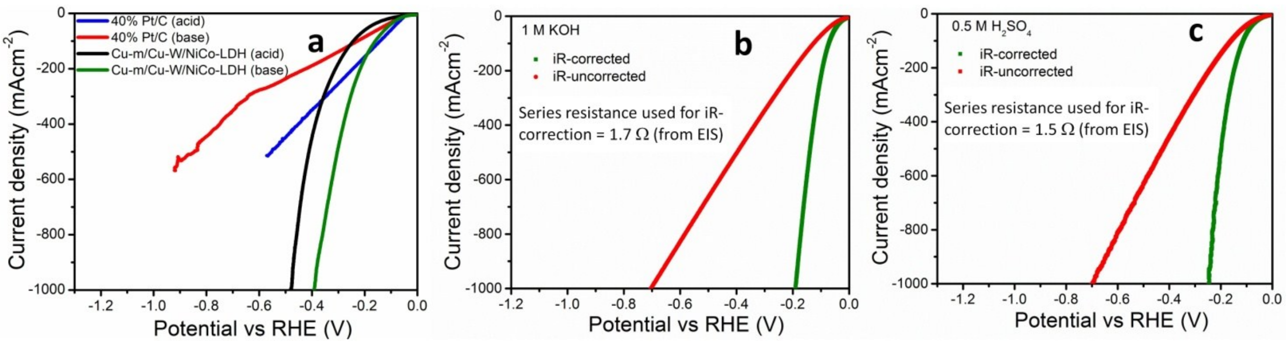

Figure S10. (a) HER performance of Cu-m/Cu-W/NiCo-LDH and 40 wt% Pt/C measured at a slow scan rate of 1 mV/s in alkaline and acidic media. LSV polarization plots of Cu-m/Cu-W/NiCo-LDH before and after *iR*-correction in (b) alkaline and (c) acidic media at 10 mV/s scan rate.

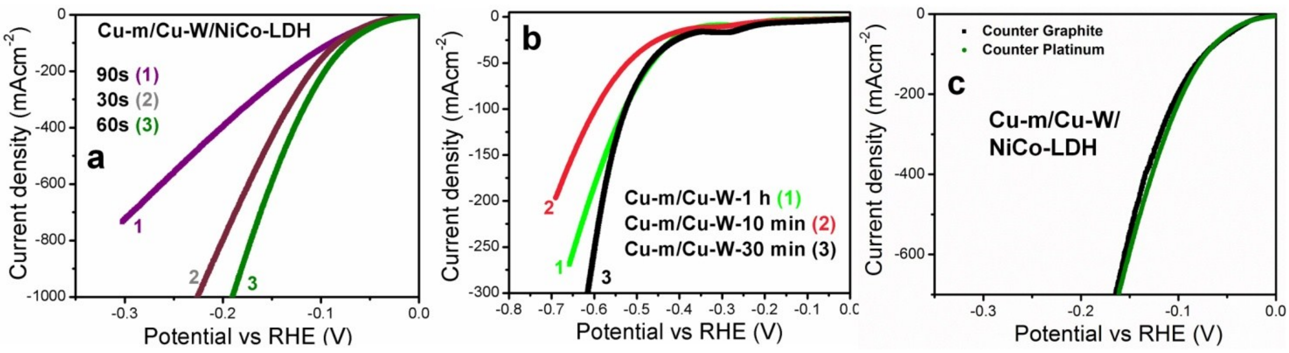

Figure S11. HER performance of Cu-m/Cu-W/NiCo-LDH electrode from control experiments in 1M KOH. LSV plots of the Cu-m/Cu-W/NiCo-LDH electrode fabricated at varying durations of (a) electrodeposition of NiCo-LDH, and (b) chemical oxidation of Cu-m. (c) HER activity of Cu-m/Cu-W/NiCo-LDH using Pt rod and graphite as counter electrodes.

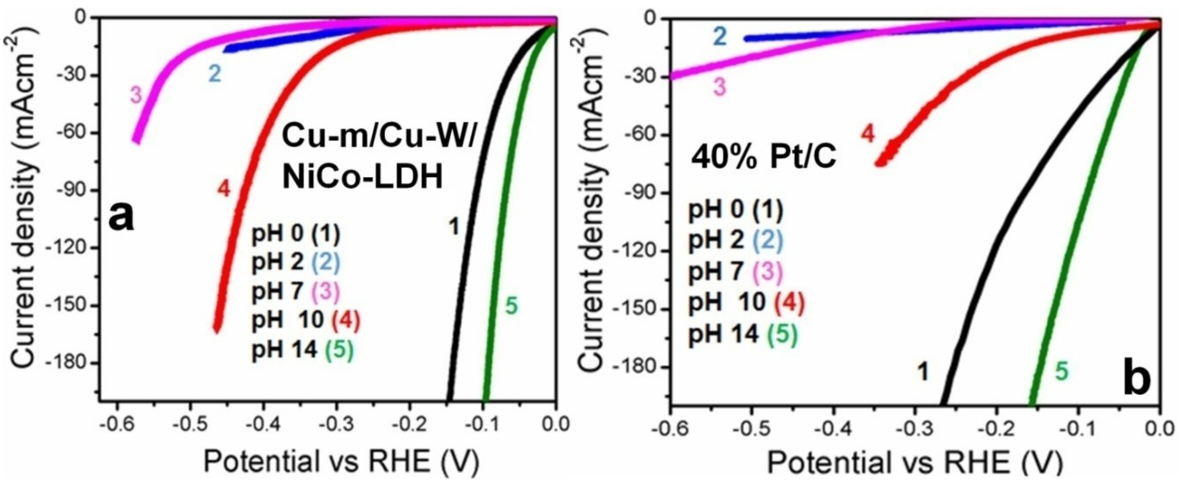

Figure S12. pH universality. LSV plots showing pH universality of (a) Cu-m/Cu-W/NiCo-LDH and (b) 40 wt% Pt/C. For each test, electrodes from different fabrication batches were used.

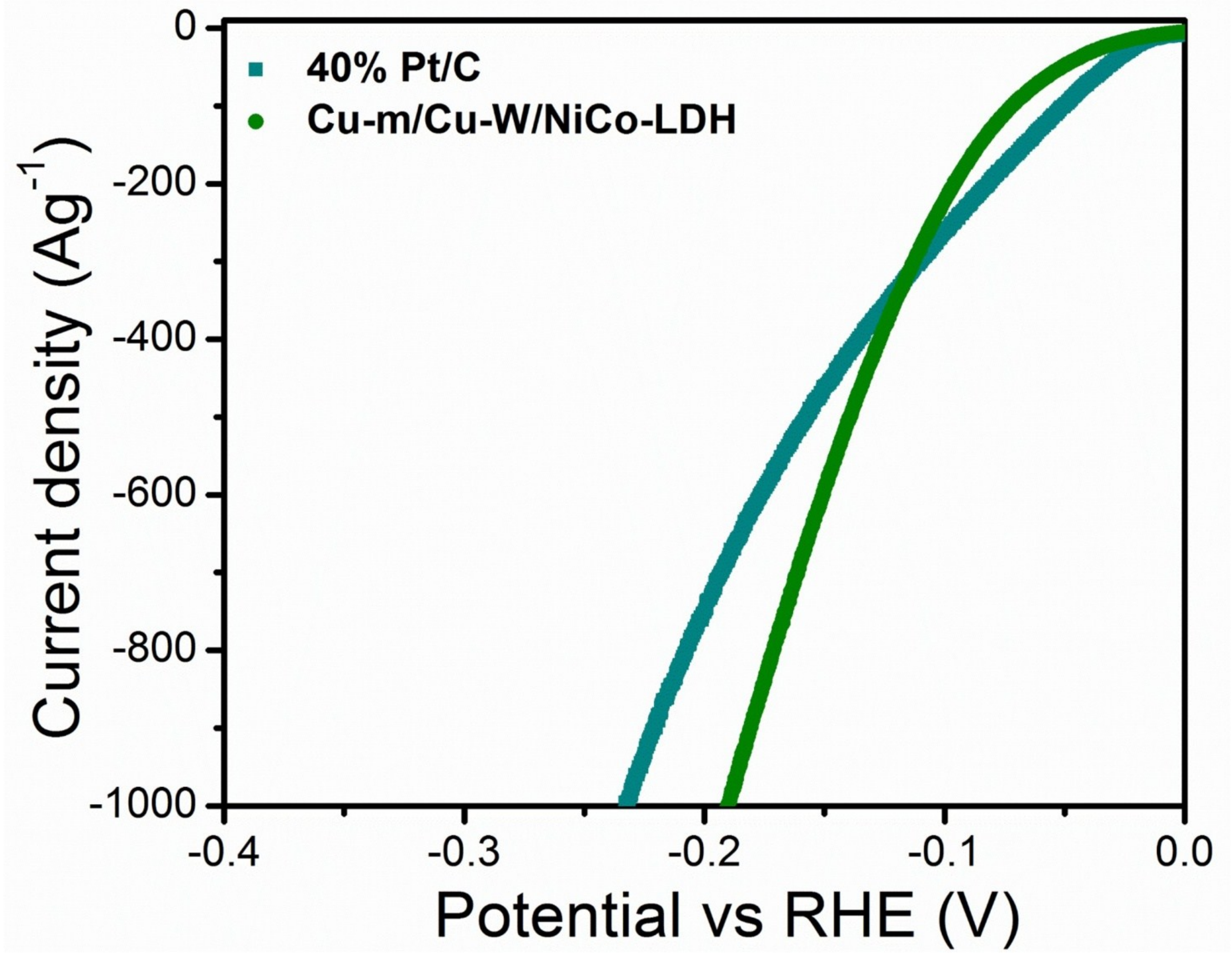

Figure S13. Mass activity of Cu-m/Cu-W/NiCo-LDH and 40 wt% Pt/C.

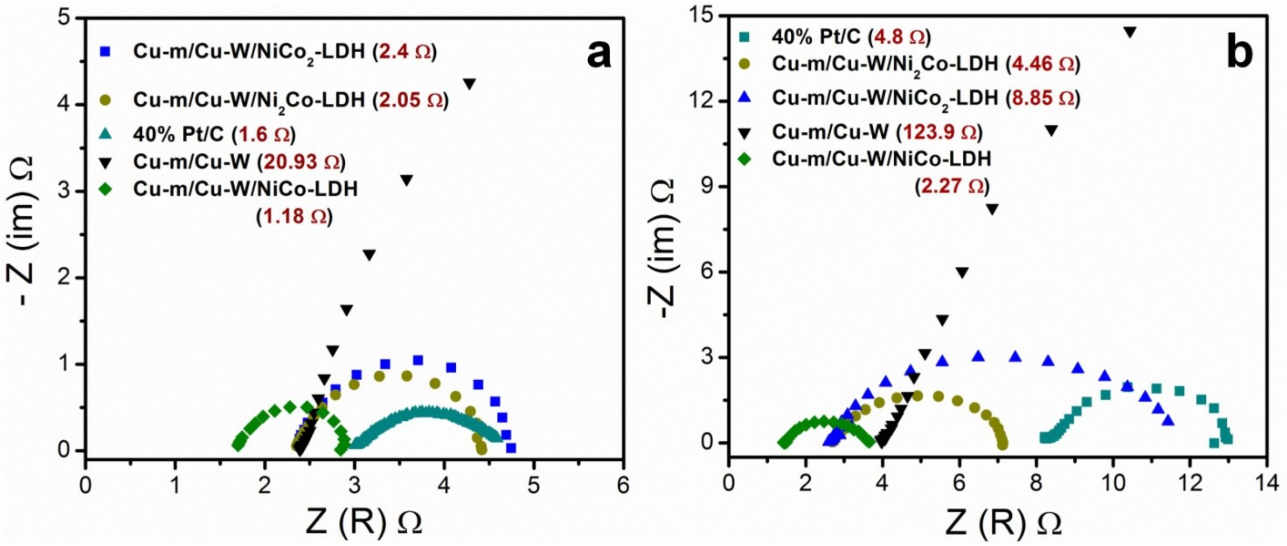

Figure S14. Nyquist plots at (a) 114 mV overpotential in 1M KOH, and (b) 190 mV overpotential in 0.5M H_2SO_4. The corresponding R_{CT} values are included within parenthesis.

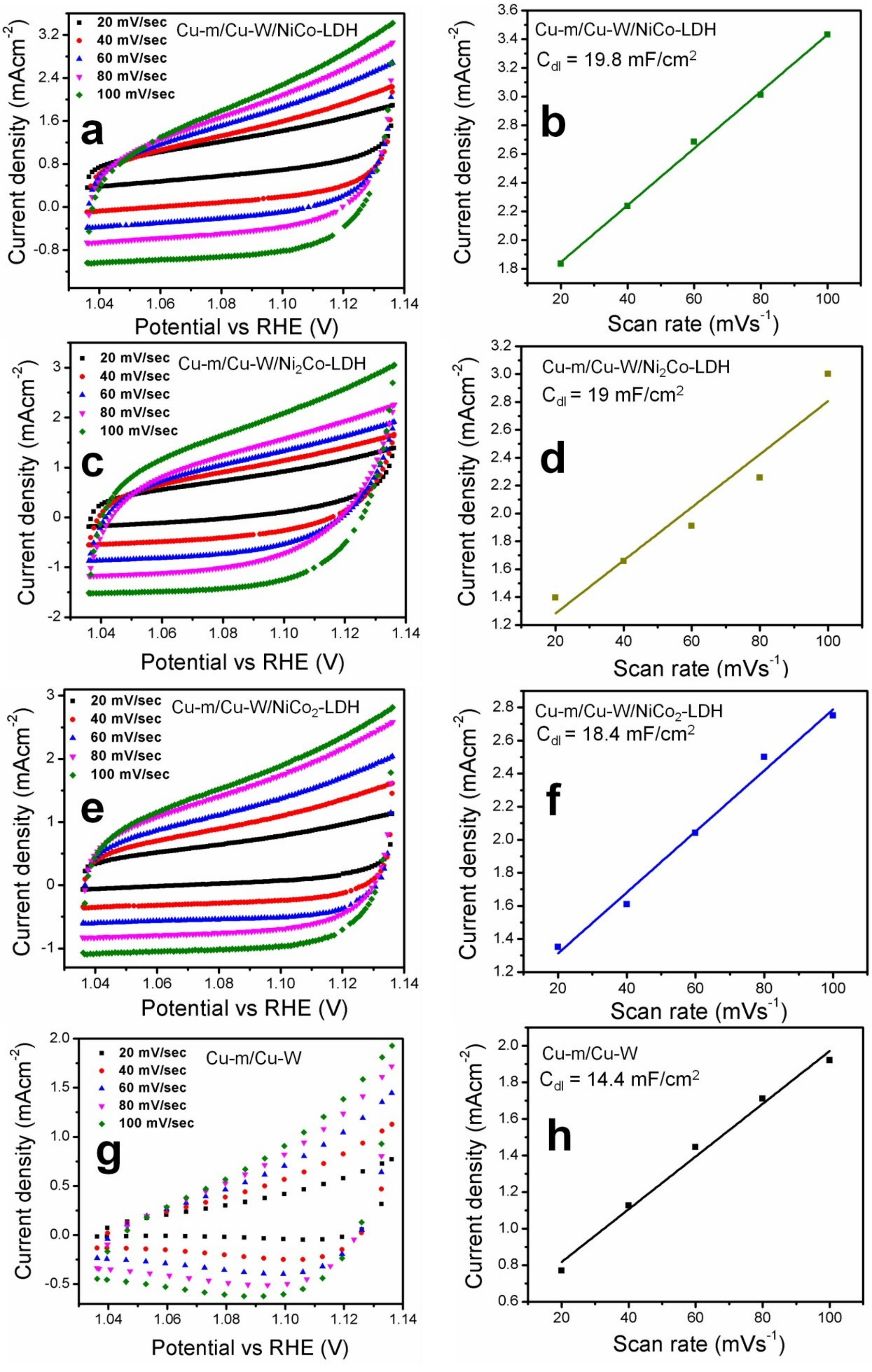

Figure S15. ECSA determination. (a, c, e, g) CV plots of Cu-m/Cu-W/NiCo-LDH, Cu-m/Cu-W/Ni_2Co-LDH, Cu-m/Cu-W/$NiCo_2$-LDH and Cu-m/Cu-W, respectively, at different scan rates. (b, d, f, h) Current density (recorded at a fixed potential) as a function of scan rate for Cu-m/Cu-W/NiCo-LDH, Cu-m/Cu-W/Ni_2Co-LDH, Cu-m/Cu-W/$NiCo_2$-LDH and Cu-m/Cu-W, respectively.

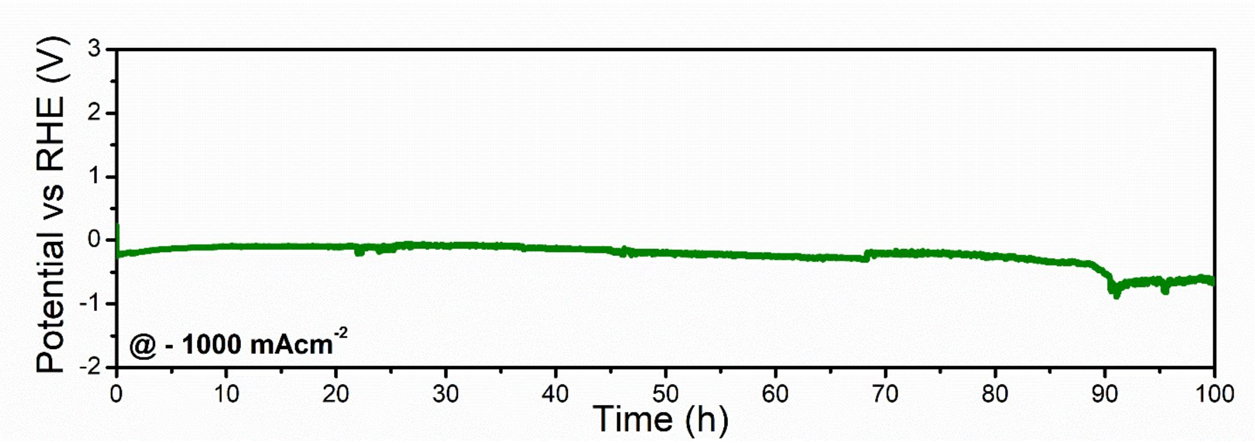

Figure S16. Chronopotentiometric stability test for 100 h at industrial high current density of -1 Acm^{-2} in 1M KOH.

Supplementary Movie 1

Incessant bubbles of hydrogen and oxygen are shown to evolve at cathode and anode, respectively, at a high current density of -1 Acm^{-2}, corroborating its potential for commercial applications.

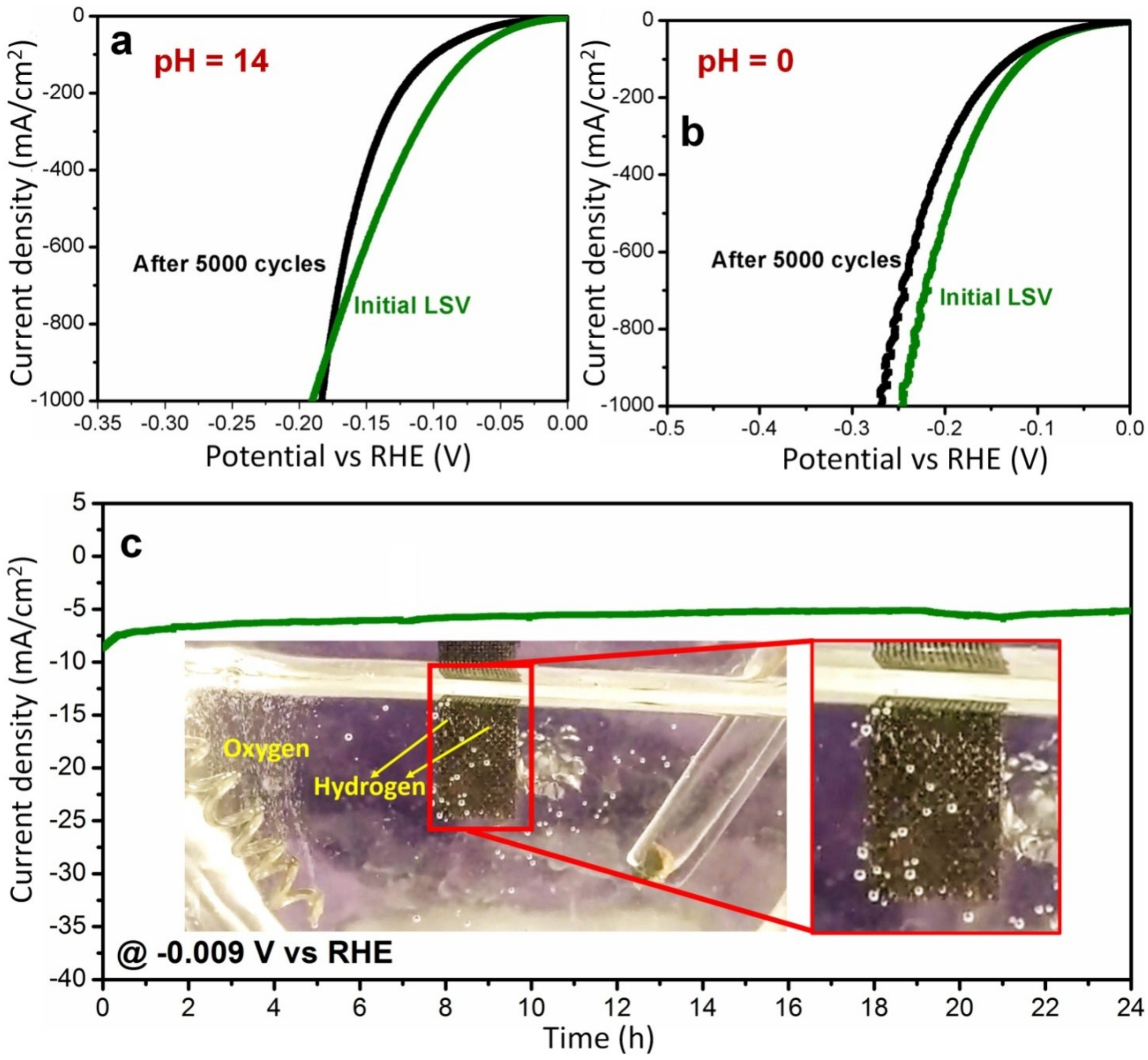

Figure S17. LSV polarization curves of Cu-m/Cu-W/NiCo-LDH electrode before and after 5000 cycles in (a) 1M KOH and (b) 0.5M H_2SO_4. (c) Stability test at low overpotential of 9 mV in 1M KOH. Inset shows the release of hydrogen bubbles at 9 mV overpotential.

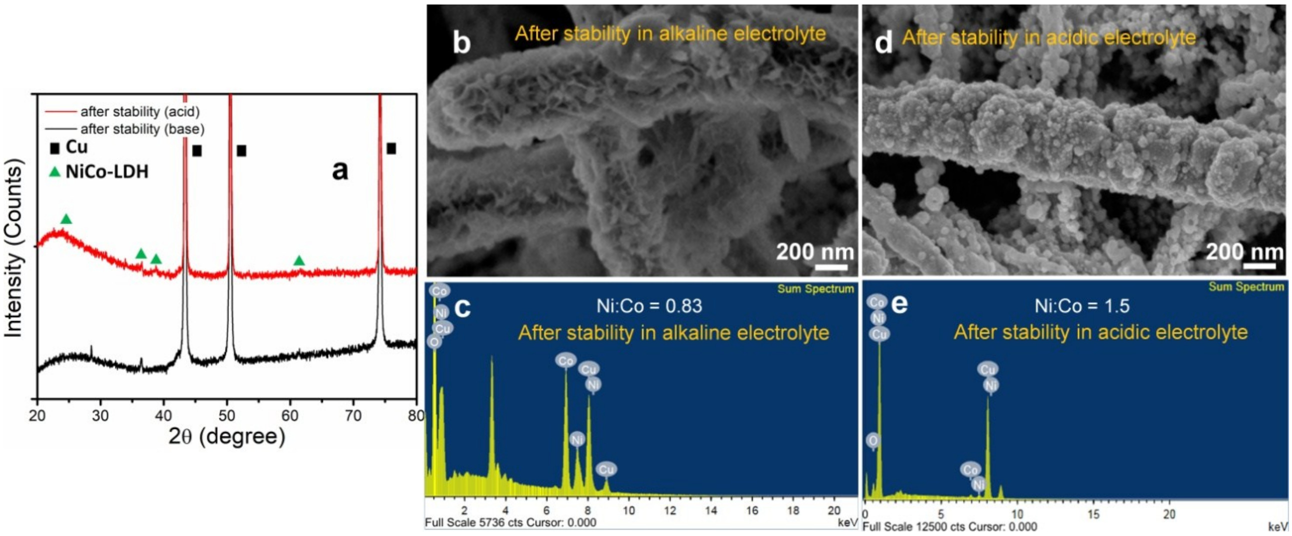

Figure S18. Characterization of the NiCo-LDH catalyst after chronopotentiometric stability tests at -1 Acm^{-2}. (a) PXRD patterns of NiCo-LDH after the stability tests in pH 14 and 0. FESEM images and EDS spectra after the stability test in (b, c) alkaline and (d, e) acidic media.

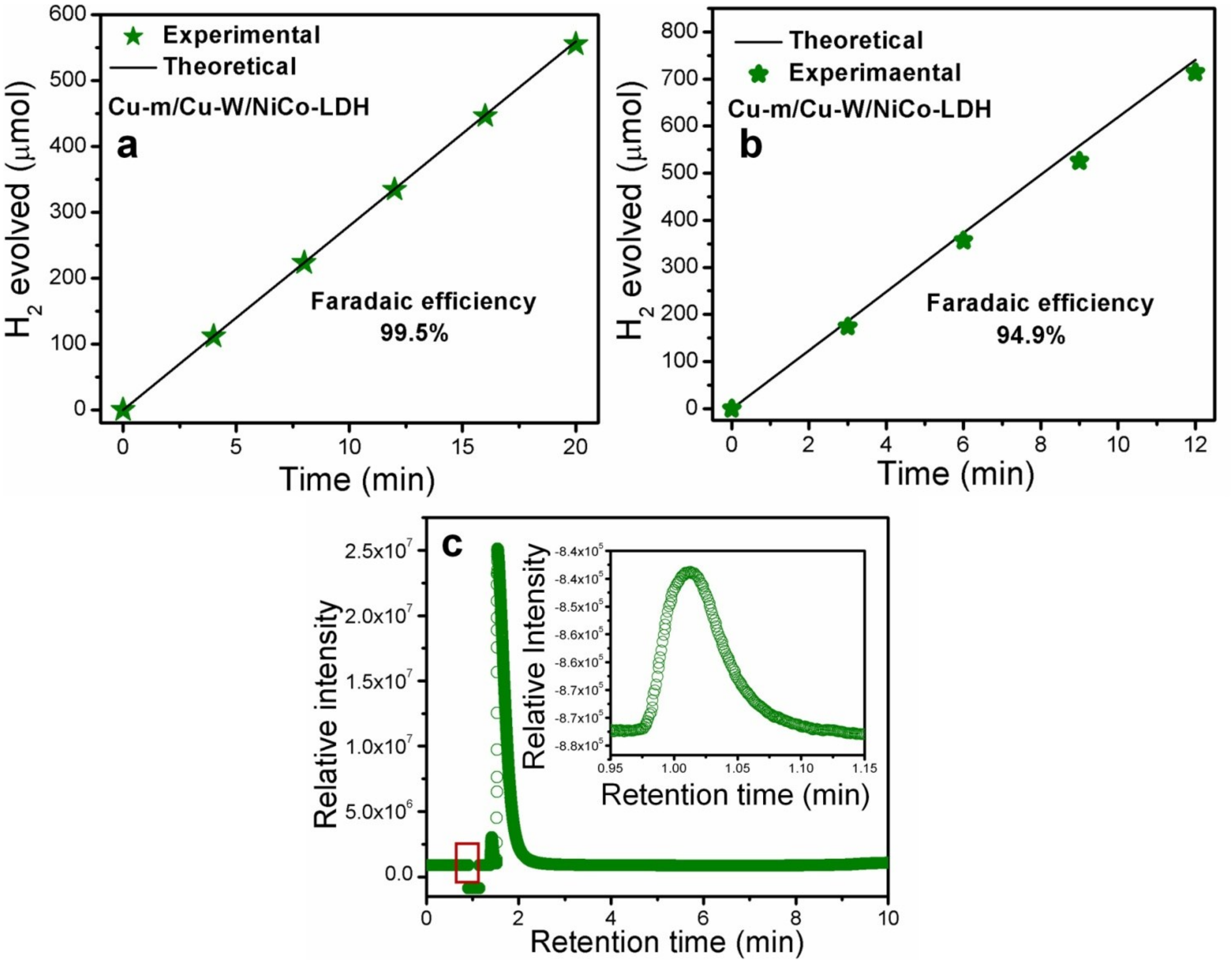

Figure S19. Faradic efficiency of Cu-m/Cu-W/NiCo-LDH electrode showing the theoretically calculated and experimentally measured H_2 gas with time in (a) 1M KOH (pH 14) and (b) 0.5 M H_2SO_4 (pH 0). (c) GC plot showing the detected H_2 gas at -1.8 V vs Ag/AgCl. Inset shows the zoom in view of the boxed area.

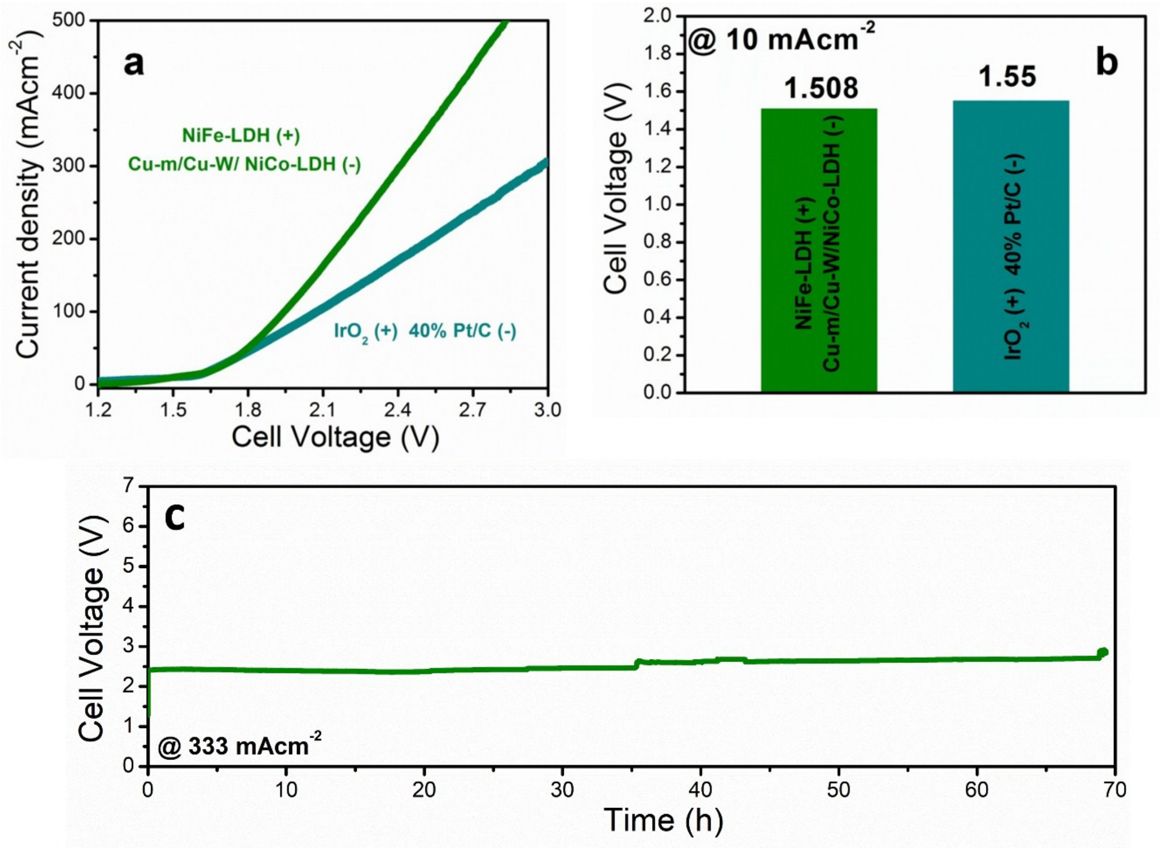

Figure S20. Overall water splitting with NiFe-LDH anode. (a) LSV polarization curves of NiFe-LDH (+) || Cu-m/Cu-W/NiCo-LDH (-) and and Pt/C (-) || IrO_2 (+) electrolyzers at a scan rate of 10 mV s^{-1}. (b) The respective cell voltages required to reach 10 $mAcm^{-2}$ current density. (c) Chronopotentiometry test of the NiFe-LDH (+) || Cu-m/Cu-W/NiCo-LDH (-) electrolyzer at 333 $mAcm^{-2}$.

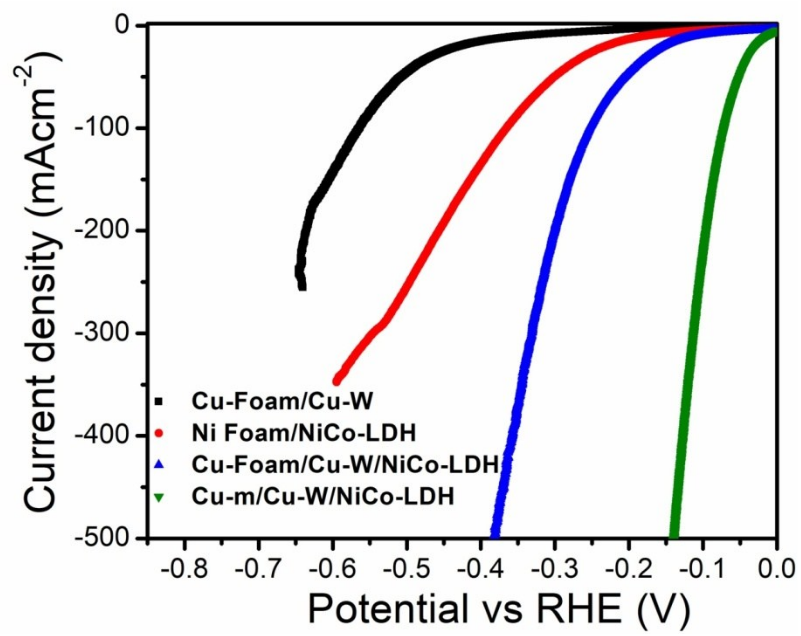

Figure S21. HER activity comparison by changing the the substrate. LSV polarization curves for Cu-foam/Cu-W, Ni-foam/NiCo-LDH, Cu-foam/Cu-W/NiCo-LDH and Cu-m/Cu-W/NiCo-LDH.

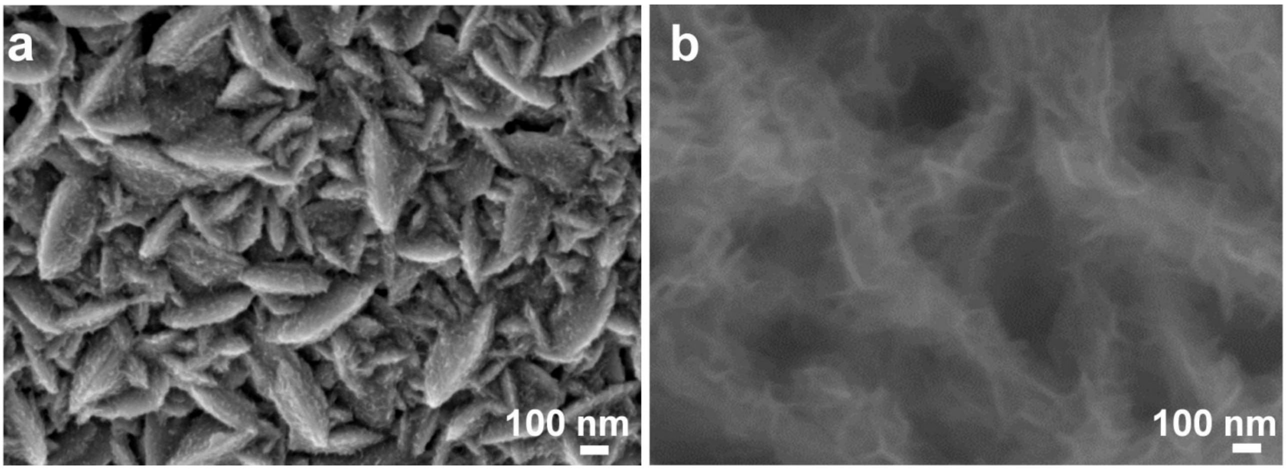

Figure S22. FESEM images of (a) Ni-foam/NiCo-LDH and (b) Cu-foam/Cu-W/NiCo-LDH.

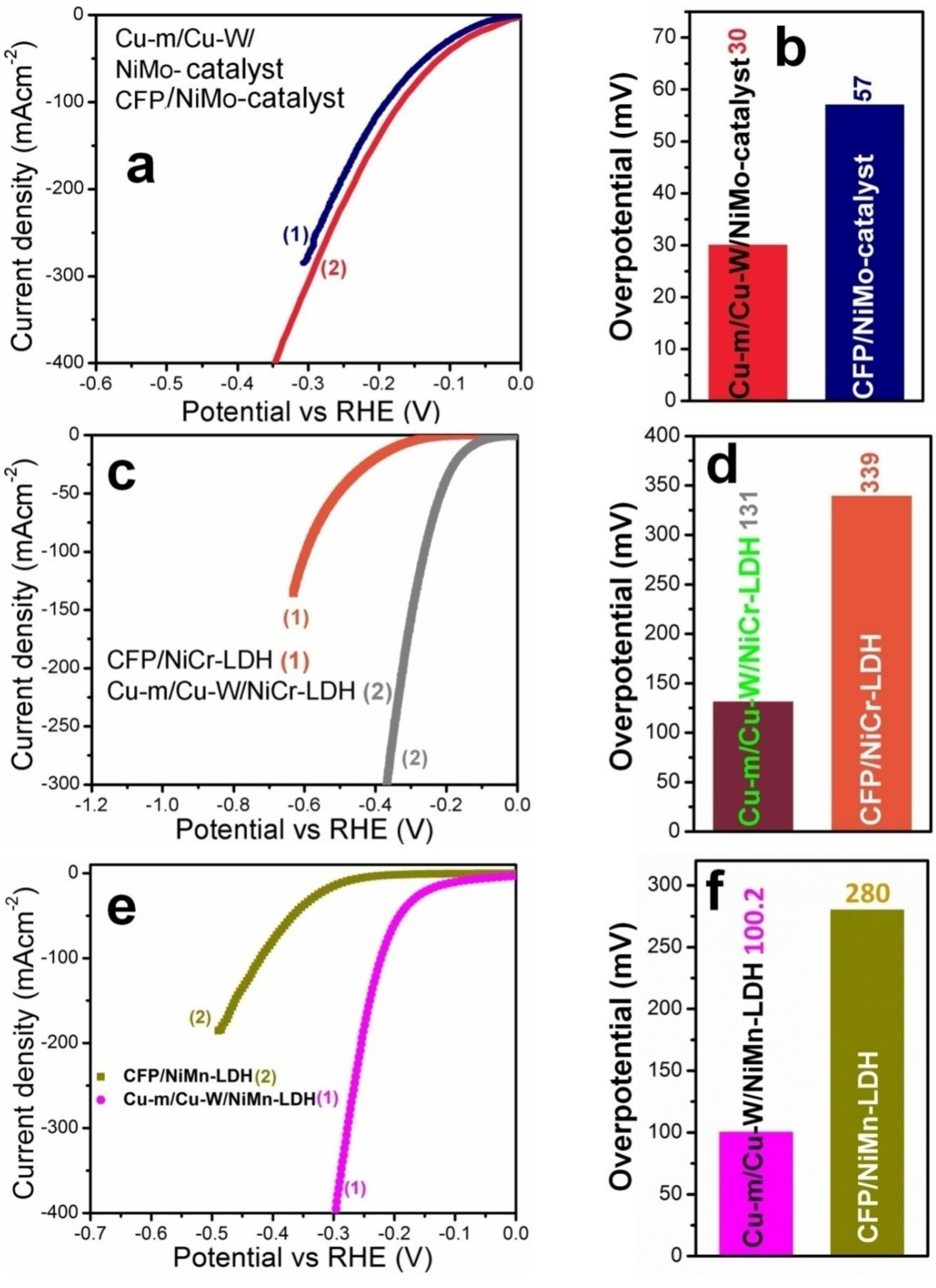

Figure S23. Choice of HER catalyst and the substrate. (a, c, e) HER activity comparison and (b, d, f) the required overpotentials to achieve -10 $mAcm^{-2}$ with NiMo-catalyst, NiCr-LDH and NiMn-LDH catalysts on Cu-m/Cu-W and CFP substrates, respectively.

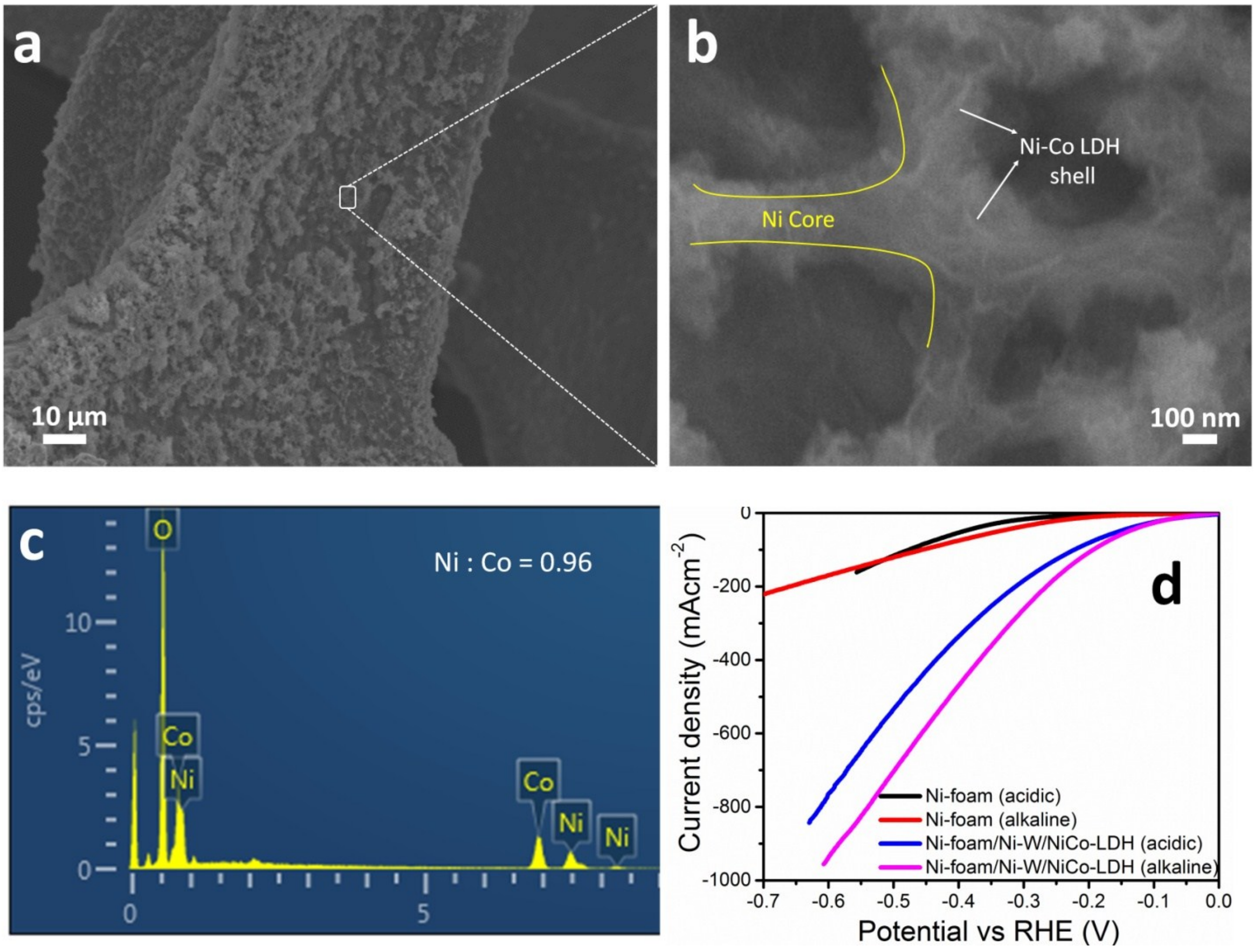

Figure S24. (a, b) FESEM images and (c) EDS spectrum of NiCo-LDH grown on Ni nanowire supported on Ni foam. (d) HER LSV plots under acidic and alkaline medium.

Figure S24a shows the rough surface of Ni foam after the growth of Ni nanowires with NiCo-LDH shell. Figure S24b clearly shows the coating of LDH on the nanowire surface, whereas the EDS spectrum confirms the presence of Ni, Co and O with Ni:Co ratio of 0.96 (Figure S24c). The LSV curves in Figure S24d show the promising performance of the NiCo-LDH coated Ni nanowire electrode with overpotential values of 48 and 52 mV at -10 mA cm^2 under acidic and alkaline media, respectively.

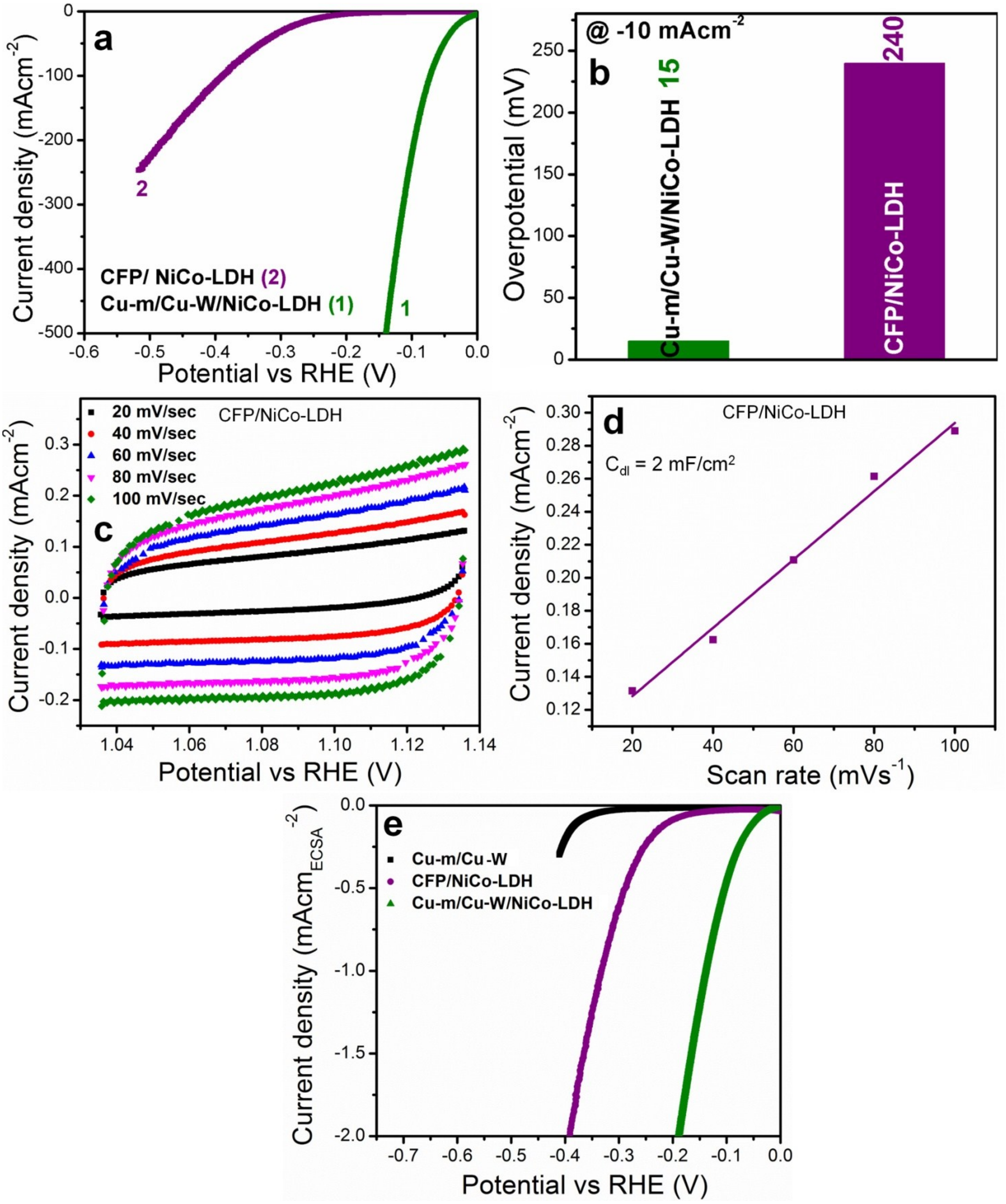

Figure S25. HER activity comparison of CFP/NiCo-LDH and Cu-m/Cu-W/NiCo-LDH. (a) LSV plots, (b) required overpotentials to achieve -10 mAcm^{-2}. (c) CV plots of CFP/NiCo-LDH at different scan rates, (d) current density (recorded at a fixed potential) as function of scan rate for CFP/NiCo-LDH. (e) Normalization of HER activity of Cu-m/Cu-W, Cu-m/Cu-W/NiCo-LDH, CFP/NiCo-LDH with respect to their corresponding ECSA (≡ C_{dl}).

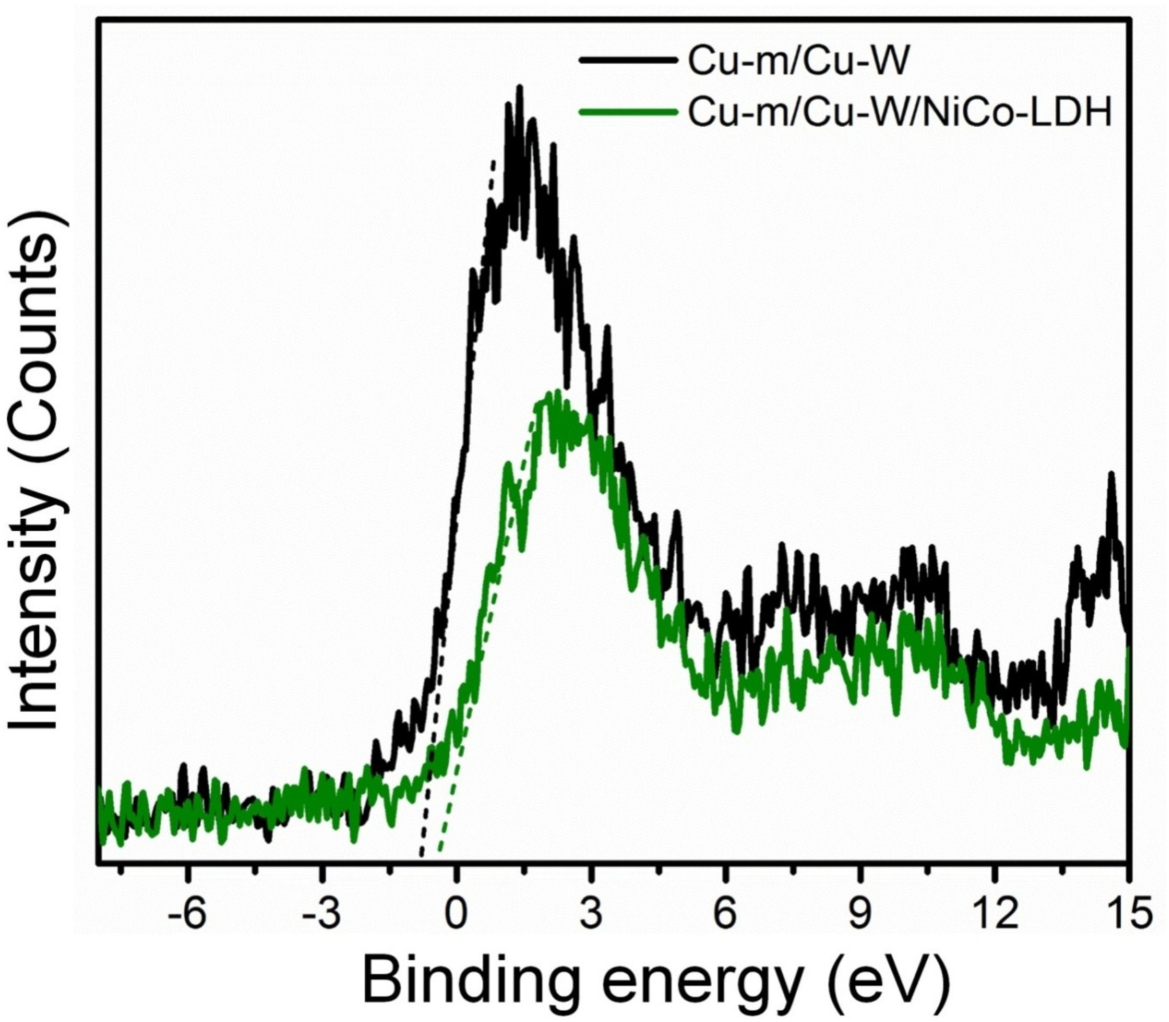

Figure S26. XPS valence band spectra of Cu-m/Cu-W and Cu-m/Cu-W/NiCo-LDH electrodes.

Table S1. Lattice parameters of different Ni_xCo_y-LDH (x:y = 1:1, 1:2, 2:1) catalysts supported on Cu-m/Cu-W substrate and the corresponding 2θ values of (006) and (110) crystallographic planes.

Catalyst	$2\theta_{(006)}$ (°)	$2\theta_{(110)}$ (°)	*c* (Å)	*a* (Å)
Cu-m/Cu-W/NiCo-LDH	23.86	58.43	22.34	3.15
Cu-m/Cu-W/$NiCo_2$-LDH	23.04	62.13	23.13	2.98
Cu-m/Cu-W/Ni_2Co-LDH	22.84	64.99	23.33	2.86

Table S2. Co:Ni molar ratios for different Ni_xCo_y-LDH (x:y = 1:1, 1:2, 2:1) catalysts supported on Cu-m/Cu-W substrate, obtained from EDX analyses, averaged over multiple locations.

Catalyst	Co (Atomic %)	Ni (Atomic %)	Co:Ni	
			Expected	Observed
Cu-m/Cu-W/NiCo-LDH	2.48 ± 0.7	2.33 ± 0.45	1:1	1.06:1
Cu-m/Cu-W/$NiCo_2$-LDH	4.05 ± 0.32	1.7 ± 0.5	2:1	2.38:1
Cu-m/Cu-W/Ni_2Co-LDH	2.7 ± 0.34	6.43 ± 0.72	1:2	1:2.38

Table S3. Overpotential (η) values of Cu-m/Cu-W/NiCo-LDH and 40 wt% Pt/C at -10 $mAcm^{-2}$ in different pH of the electrolyte.

Cu-m/Cu-W/NiCo-LDH		40 wt% Pt/C	
pH	η_{10} (mV)	pH	η_{10} (mV)
0	27	0	18
2	334	2	503
7	443	7	387.6
10	273.7	10	132
14	15	14	22

Table S4. Comparison table of the HER performance of Cu-m/Cu-W/NiCo-LDH electrode with reported electrocatalysts in alkaline medium (pH = 14).

Catalyst	Overpotential (mV)	Ref.
Cu-m/Cu-W/NiCo-LDH	15 mV @ -10 $mAcm^{-2}$<br>72 mV @ -100 $mAcm^{-2}$	This work
40 wt% Pt/C	22 mV @ -10 $mAcm^{-2}$<br>96.9 mV @ -100 $mAcm^{-2}$	This work
$Ru/W_{0.62}(N_{0.62}O_{0.38})@C$	2 mV @ -10 $mAcm^{-2}$<br>57 mV @ -100 $mAcm^{-2}$	S1
Ir@cage-like organic network	13.6 mV @ -10 $mAcm^{-2}$	S2
CoRu	13 mV @ -10 $mAcm^{-2}$	S3
Ru@nitrogenated holey two-dimensional carbon	17 mV @ -10 $mAcm^{-2}$<br>35.5 mV @ -20 $mAcm^{-2}$	S4
Small Ru phosphide / C alloy	17 mV @ -10 $mAcm^{-2}$<br>71 mV @ -100 $mAcm^{-2}$	S5
Ru-Co	28 mV @ -10 $mAcm^{-2}$<br>218 mV @ -100 $mAcm^{-2}$	S6
RuP_2/N, P-doped carbon	52 mV @ -10 $mAcm^{-2}$<br>110 mV @ -50 $mAcm^{-2}$	S7
hcp PtNi	65 mV @ -10 $mAcm^{-2}$	S8
CoP/Ni_5P_4	71 mV @ -10 $mAcm^{-2}$<br>140 mV @ -100 $mAcm^{-2}$	S9
NiO/Ni/CNT	86 mV @ -10 $mAcm^{-2}$<br>100 mV @ -20 $mAcm^{-2}$	S10
5:95 Ag:Ni heterogeneous alloy	24mV @ -10 $mAcm^{-2}$<br>125mV @ -20 $mAcm^{-2}$	S11

Table S5. Comparison table of the HER performance of Cu-m/Cu-W/NiCo-LDH electrode with reported electrocatalysts in acidic medium (pH = 0).

Catalyst	Overpotential (mV)	Ref.
Cu-m/Cu-W/NiCo-LDH	27 mV @ -10 $mAcm^{-2}$<br>112 mV @ -100 $mAcm^{-2}$	This work
40 wt% Pt/C	18 mV @ -10 $mAcm^{-2}$<br>180 mV @ -100 $mAcm^{-2}$	This work
Ir@cage-like organic network	13.6 mV @ -10 $mAcm^{-2}$	S2
RuPt	19.7 mV @ -10 $mAcm^{-2}$<br>43.1 mV @ -100 $mAcm^{-2}$	S12
Ru@nitrogenated holey two-dimensional carbon	22 mV @ -10 $mAcm^{-2}$<br>50 mV @ -30 $mAcm^{-2}$	S4
Pt-MXene	30 mV @ -10 $mAcm^{-2}$<br>77 mV @ -100 $mAcm^{-2}$	S13
CoP/Ni_5P_4	33 mV @ -10 $mAcm^{-2}$<br>85 mV @ -100 $mAcm^{-2}$	S9
RuP_2/ N, P-doped carbon	38 mV @ -10 $mAcm^{-2}$<br>50 mV @ -50 $mAcm^{-2}$	S7
CoP/polyaniline	71 mV @ -10 $mAcm^{-2}$<br>125 mV @ -100 $mAcm^{-2}$	S14
IrHNC	4.5 mV @ -10 $mAcm^{-2}$<br>39 mV @ -100 $mAcm^{-2}$	S15

Discussion S1. TOF calculation.

TOFs for HER in alkaline medium (pH = 14):

The number of hydrogen turnover is calculated from the current density according to:

Number of hydrogen turnover = $\left(j\frac{mA}{cm^2}\right)\left(\frac{1\ C/s}{1000\ mA}\right)\left(\frac{1\ mol\ e-}{96485.3\ C}\right)\left(\frac{1\ mol\ H_2}{2\ mol\ e-}\right) \times (6.023 * 10^{23})$

<u>Cu-m/Cu-W/NiCo-LDH</u>

At an overpotential of 30 mV, Cu-m/Cu-W/NiCo-LDH reached a current density of 21.1 $mAcm^{-2}$.

Number of hydrogen turnover for Cu-m/Cu-W/NiCo-LDH at 30 mV overpotential:

$$\left(21.1\frac{mA}{cm^2}\right)\left(\frac{1\ C/s}{1000\ mA}\right)\left(\frac{1\ mol\ e-}{96485.3\ C}\right)\left(\frac{1\ mol\ H_2}{2\ mol\ e-}\right) \times (6.023 \times 10^{23}) = 6.584 \times 10^{16}\left(\frac{1/s}{cm^2}\right)$$

On the basis of ECSA along with the unit cell (volume of 221.66 Å^3) of the NiCo-LDH crystal structure in Cu-m/Cu-W/NiCo-LDH, active sites per real surface area:

Active sites = $\left(\frac{2\ atom/unit\ cell}{221.66\ Å^3/unit\ cell}\right)^{2/3} = 0.043\times10^{16} atoms\ cm^{-2}_{real}$

TOF (η_{30}) = $\frac{6.584\ x\ 10^{16}\left(\frac{1/s}{cm^2}\right)}{Surface\ sites\ \times\ ECSA} = 0.3\ s^{-1}$

TOF of Cu-m/Cu-W/NiCo-LDH for HER is 0.3 s^{-1} at an overpotential of 30 mV.

<u>40 wt% Pt/C</u>

At an overpotential of 30 mV, 40 wt% Pt/C reached a current density of 17.8 mA cm^{-2}.

Number of hydrogen turnover for 40 wt% Pt/C at 30 mV overpotential:

$$\left(17.8\frac{mA}{cm^2}\right)\left(\frac{1\ C/s}{1000\ mA}\right)\left(\frac{1\ mol\ e-}{96485.3\ C}\right)\left(\frac{1\ mol\ H_2}{2\ mol\ e-}\right) \times (6.023 * 10^{23}) = 5.55 \times 10^{16}\left(\frac{1/s}{cm^2}\right)$$

On the basis of ECSA along with the unit cell (volume of 60.23 Å^3) of the cubic Pt crystal structure in the 40 wt% Pt/C. Active sites per real surface area:

Active sites = $\left(\frac{4\ atom/unit\ cell}{60.23\ Å^3/unit\ cell}\right)^{2/3} = 0.164 \times 10^{16} atoms\ cm^{-2}_{real}$

TOF (η_{30}) = $\frac{5.55\ \times\ 10^{16}\left(\frac{1/s}{cm^2}\right)}{Surface\ sites\ *\ ECSA} = 0.017\ s^{-1}$

TOF of 40 wt% Pt/C for HER is 0.017 s^{-1} at an overpotential of 30 mV.

TOFs for HER in acidic medium (pH = 0):

Cu-m/Cu-W/NiCo-LDH

At an overpotential of 30 mV, Cu-m/Cu-W/NiCo-LDH reached a current density of 11.25 mA cm^{-2}.

Number of hydrogen turnover for Cu-m/Cu-W/NiCo-LDH at 30 mV overpotential:

$$\left(11.25\frac{mA}{cm^2}\right)\left(\frac{1\ C/s}{1000\ mA}\right)\left(\frac{1\ mol\ e-}{96485.3\ C}\right)\left(\frac{1\ mol\ H_2}{2\ mol\ e-}\right)*(6.023*10^{23}) = 3.511\ \text{x}\ 10^{16}\left(\frac{1/s}{cm^2}\right)$$

On the basis of ECSA along with the unit cell (volume of 221.66 Å^3) of the NiCo-LDH crystal structure in the Cu-m/Cu-W/NiCo-LDH. Active sites per real surface area:

$$\text{Active sites} = \left(\frac{2\ atom/unit\ cell}{221.66\ \text{Å}^3/unit\ cell}\right)^{2/3} = 0.043\times10^{16} atoms\ cm^{-2}{}_{real}$$

$$\text{TOF}\ (\eta_{30}) = \frac{3.511\ x\ 10^{16}\left(\frac{1/s}{cm^2}\right)}{Surface\ sites\ \times\ ECSA} = 0.317\ s^{-1}$$

TOF of Cu-m/Cu-W/NiCo-LDH for HER is 0.317 s^{-1} at an overpotential of 30 mV.

40 wt% Pt/C

At an overpotential of 30 mV, 40 wt% Pt/C reached a current density of 14.28 mA cm^{-2}.

Number of hydrogen turnover for 40 wt% Pt/C at 30 mV overpotential:

$$\left(14.28\frac{mA}{cm^2}\right)\left(\frac{1\ C/s}{1000\ mA}\right)\left(\frac{1\ mol\ e-}{96485.3\ C}\right)\left(\frac{1\ mol\ H_2}{2\ mol\ e-}\right)\times(6.023*10^{23}) = 4.45\times 10^{16}\left(\frac{1/s}{cm^2}\right)$$

On the basis of ECSA along with the unit cell (volume of 60.23 Å^3) of the cubic platinum crystal structure in the 40 wt% Pt/C. Active sites per real surface area:

$$\text{Active sites} = \left(\frac{4\ atom/unit\ cell}{60.23\ \text{Å}^3/unit\ cell}\right)^{2/3} = 0.164\times10^{16} atoms\ cm^{-2}{}_{real}$$

$$\text{TOF}\ (\eta_{30}) = \frac{4.45\ x\ 10^{16}\left(\frac{1/s}{cm^2}\right)}{Surface\ sites\ \times\ ECSA} = 0.01\ s^{-1}$$

TOF of 40 wt% Pt/C for HER is 0.01 s^{-1} at an overpotential of 30 mV.

Supplementary References

S1. L. Zhang, Z. Lang, Y. Wang, H. Tan, H. Zang, Z. Kang, Y. Li, *Energy Environ. Sci.*, 2019*,* **12**, 2569-2580.

S2. J. Mahmood, M. Anjum, S. Shin, I. Ahmad, H. Noh, S. Kim, H. Jeong, J. S. Lee, J. B. Baek, *Adv. Mater.*, 2018, **30**, 1805606.

S3. J. Mao, C. He, J. Pei, W. Chen, D. He, Y. He, Z. Zhuang, C. Chen, Q. Peng, D. Wang, Y. Li, *Nat. Commun.*, 2018**, 9**, 4958.

S4. J. Mahmood, F. Li, S. Jung, M. Okyay, I. Ahmad, S. Kim, N. Park, H. Jeong, J. Baek, *Nat. Nanotechnol.* 2017, **12**, 441-446.

S5. J. Yu, Y. Guo, S. She, S. Miao, M. Ni, W. Zhou, M. Liu, Z. Shao, *Adv. Mater.,* 2018, **30**, 1800047.

S6. J. Su, Y. Yang, G. Xia, J. Chen, P. Jiang, Q. Chen, *Nat. Commun.,* 2017, **8**, 16028.

S7. Z. Pu, I. Amiinu, Z. Kou, W. Li, S. Mu, *Angew. Chem. Int. Ed*. 2017, **129**, 11717-11722.

S8. Z. Cao, Q. Chen, J. Zhang, H. Li, Y. Jiang, S. Shen, G. Fu, B. Lu, Z. Xie, L. Zheng, *Nat. Commun.*, 2017, **8**, 15131.

S9. I. Mishra, H. Zhou, J. Sun, F. Qin, K. Dahal, J. Bao, S. Chen, Z. Ren, *Energy & Environ. Sci.,* 2018, **11**, 2246-2252.

S10. M. Gong, W. Zhou, M. Tsai, J. Zhou, M. Guan, M. Lin, B. Zhang, Y. Hu, D. Wang, J. Yang, S. Pennycook, B. Hwang, H. Dai, *Nat Commun.*, 2014, **5**, 4695.

S11. R. Majee, A. Kumar, T. Das, S. Chakraborty, S. Bhattacharyya, *Angew. Chem. Int. Ed.*, 2019, **59**, 2881-2889.

S12. K. Li, Y. Li, Y. Wang, J. Ge, C. Liu, W. Xing, *Energy Environ. Sci.*, 2018*,* **11**, 1232-1239.

S13. J. Zhang, Y. Zhao, X. Guo, C. Chen, C. Dong, R. Liu, C. Han, Y. Li, Y. Gogotsi, G. Wang, *Nat. Catal.* 2018, **1**, 985-992.

S14. J. Feng, S. Tong, Y. Tong, G. Li, *J. Am. Chem. Soc.*, 2018, **140**, 5118-5126.

S15. F. Li, G. Han, H. Noh, J. Jeon, I. Ahmad, S. Chen, C. Yang, Y. Bu, Z. Fu, Y. Lu, *Nat. Commun.*, 2019, **10**, 4060.
